# The lipid-dependent structure and function of LacY can be recapitulated and analyzed in phospholipid-containing detergent micelles

**DOI:** 10.1038/s41598-019-47824-y

**Published:** 2019-08-05

**Authors:** Heidi Vitrac, Venkata K P S Mallampalli, Mikhail Bogdanov, William Dowhan

**Affiliations:** Department of Biochemistry and Molecular Biology and the Center for Membrane Biology, University of Texas McGovern Medical School at Houston, Houston, TX 77030 USA

**Keywords:** Membrane lipids, Membrane proteins

## Abstract

Membrane proteins play key roles in cellular functions, their activity mainly depending on their topological arrangement in membranes. Structural studies of membrane proteins have long adopted a protein-centric view regarding the determinants of membrane protein topology and function. Several studies have shown that the orientation of transmembrane domains of polytopic membrane proteins with respect to the plane of the lipid bilayer can be largely determined by membrane lipid composition. However, the mechanism by which membrane proteins exhibit structural and functional duality in the same membrane or different membranes is still unknown. Here we show that lipid-dependent structural and functional assessment of a membrane protein can be conducted in detergent micelles, opening the possibility for the determination of lipid-dependent high-resolution crystal structures. We found that the lactose permease purified from *Escherichia coli* cells exhibiting varied phospholipid compositions exhibits the same topology and similar function as in its membrane of origin. Furthermore, we found several conditions, including protein mutations and micelle lipid composition, that lead to increased protein stability, correlating with a higher yield of two-dimensional crystal formation. Altogether, our results demonstrate how the membrane lipid environment influences membrane protein topology and arrangement, both in native membranes and in mixed detergent micelles.

## Introduction

Membrane proteins represent about 40% of the proteome and are essential components of many indispensable biological processes. Determination of high-resolution structures of membrane proteins is therefore crucial for understanding normal cell function and for the development of pharmacological modulation strategies. How lipid-protein interactions affect membrane protein structural organization is uniquely important to this group of proteins. Membrane lipid composition is dynamic during the cell cycle within a given membrane^1^ and unique to each membrane within a cell^2^. Lipid chemical and compositional diversity, both laterally within a leaflet and across the bilayer, brings an additional level of complexity to the lipid-dependent membrane protein structure and function. Therefore, how changes in membrane protein lipid environment within a given membrane or during trafficking of proteins between membranes affects protein structure must be addressed.

In order to undertake structural studies on membrane proteins, solubilization of the target proteins has to be achieved. Therefore, many successful structural studies of membrane proteins are conducted in detergent solutions. A role for membrane protein lipid environment in determining membrane protein organization has been minimized mainly due to proper folding of many membrane proteins in detergents^3^; however, why some membrane proteins misfold in detergents or when expressed in a foreign host has not been fully explained. In most cases significant amounts of lipid remain associated with the proteins and extensive delipidation may destabilize the target protein^4–6^. The often-deleterious effects of detergents on membrane protein stability, function, and structure are increasingly acknowledged^7,8^. There are only a few studies that have systematically addressed how lipid environment affects high-resolution structural determination of membrane proteins^9–11^. Despite significant progress made in the field of membrane protein structural determination (including crystallization in lipidic cubic phase^12^ and 2D electron crystallography^13,14^), lipids are most often simply considered as a parameter for optimizing crystallization rather than a necessary component of the native membrane protein environment.

There is a crucial need to better understand the impact of a defined or changing membrane lipid composition in which a membrane protein of interest is embedded. An increasing number of reports indicate that several membrane proteins display different orientations in different cellular organelles, exist in the same membrane in multiple topological orientations, or exhibit lipid-dependent function^15–22^. Therefore, how different native lipid environments affect protein structure is important not only for this subset of proteins but for many proteins thought to be immune from changes in their hydrophobic environment.

**Figure 1:**
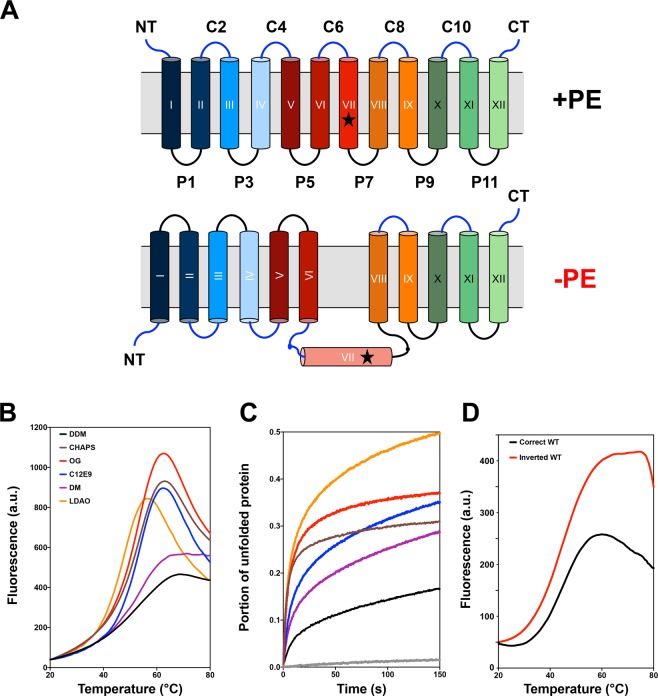
Inverted LacY stability in various detergents as quantified by the CPM dye binding assay. (**A**) Topological organization of LacY purified from PE-containing and PE-lacking cells. Cytoplasm is at the top and cylinders indicate TMDs numbered sequentially in Roman numerals from the N-terminal (NT) to the C-terminal (CT) domain. EMDs connecting the TMDs are numbered sequentially and prefixes C and P stand for cytoplasmic and periplasmic, respectively. The star symbol depicts the position of single cysteine replacement I230C in TMD VII. (**B**) Representative melting curves and (**C**) representative unfolding curves at 45 °C over the course of 150 min for WT LacY purified from PE-lacking cells and solubilized in 3xCMC of various detergents prior to the assay. LacY was diluted to 26 μg/mL in 50 mM Tris-HCl (pH 7.5), 100 mM NaCl containing 0.05% of the following detergents: CHAPS, OG, LDAO, C12E9, DM and DDM. (**D**) Melting curves for WT LacY purified from PE-containing (correct topology) and PE-lacking (inverted topology) cells. LacY was diluted to 50 μg/mL in 50 mM Tris-HCl (pH 7.5), 100 mM NaCl containing 0.05% DDM.

Dogma dictates that membrane protein topology is determined and fixed at the time of initial assembly^23–26^. Using several secondary transporters found in *Escherichia coli* including lactose permease (LacY) as model membrane proteins, we challenged this long-standing protein-centric dogma by demonstrating that the topological organization of a membrane protein, once established, is not static but is dynamic in response to changes in the lipid environment both *in vivo* and *in vitro*^27–33^. LacY, when initially assembled *in vivo* or reconstituted into proteoliposomes in its native phospholipid environment of 70–80 percent zwitterionic phosphatidylethanolamine (PE), with the remainder being anionic phosphatidylglycerol (PG) plus cardiolipin (CL), assembles as a 12 transmembrane domain (TMD) integral membrane protein consisting of two domains of six TMDs (Fig. 1A). However, when inserted into cell membranes or proteoliposomes lacking PE, LacY assumes an inverted topology with the N-terminal 6-TMD helical bundle inverted with respect to the C-terminal 5-TMD bundle and the plane of the lipid bilayer (Fig. 1A). In addition, TMD VII is now exposed as an extramembrane domain (EMD) and acts as a hinge point between the two TMD bundles. Membrane proteins can also undergo TMD flipping after initial assembly in response to changes in the lipid environment *in vivo*^29,34–37^ and *in vitro*^38–41^. Remarkably, these two topological conformers are fully interconvertible post-assembly by the addition or deletion of PE. These conformers stably co-exist in membranes with the proportion of each dependent on the level of PE in the membrane. Thus, membrane protein topological organization is not static but highly dynamic.

Successful crystallization of native LacY was first obtained in 2003 using a mutant of increased stability (C154G), allowing the precise depiction of the network of residue-residue interactions within the substrate translocation pathway of the central cavity^42^. Several of these residues are located in TMDs from the NT-bundle, which is inverted in LacY purified from PE-lacking cells. To date all conclusions on the role of lipids in determining the structure of LacY are based on gross measurements of TMD topological orientation using the substituted cysteine accessibility method as applied to TMD determination (SCAM^TMD^)^43^. More refined structural information is clearly needed regarding the organization of LacY as a function of the lipid environment in order to establish the underlying molecular basis for the dynamic properties of membrane proteins in general.

Since proteins and lipids have co-evolved and depend on each other for their respective biological functions, it is crucial to study and characterize membrane protein structure under conditions that closely resemble their native lipid environment. The overall goal of this work is to acquire molecular details of lipid-dependent effects on structure and function of LacY. To do so, we sought to determine the optimal conditions leading to the generation of crystals suitable for 2D electron crystallographic determination of structures of LacY as a function of the composition of the lipid environment. In this report we establish that LacY topological organization in detergent extracts reflects that observed in cell membrane as a function of the lipid environment. In addition, we present new structural information on the arrangement, stability and properties of inverted LacY after extraction from cells lacking PE.

## Results

### Assessing the stability of the inverted LacY in detergent solutions

LacY from *Escherichia coli* is a paradigm for secondary transporters throughout nature and has thus been heavily studied for many years^44^. Despite the many advances made regarding the structure and function of LacY using a variety of biochemical and biophysical techniques, LacY remains a very challenging protein to crystallize, leading to a large body of literature assessing the optimal conditions for LacY stability and crystallization. Several studies pointed out the strong detergent dependency of LacY’s behavior in the solubilized state^45–47^, which established that dodecyl maltoside (DDM) is the most suitable detergent, both for purification and crystallization trials, ultimately leading to multiple high-resolution determinations of LacY structure^42,48–51^. Further, it was shown that excess delipidation of LacY due to multiple chromatographic purification steps leads to the generation of poorly diffracting crystals, highlighting the importance of maintaining some level of native membrane environment to maintain LacY’s stability^48^.

The inverted topology of LacY observed in cells and proteoliposomes lacking the major membrane phospholipid PE (Fig. 1A) exhibits a similar yet different architecture compared to the wild type (WT) LacY and should thus be considered as a conformationally and topologically different yet related protein, posing significant new challenges for crystallization. Previous work from our group established that DDM was a suitable detergent for inverted LacY purification^39,40^, but no detergent stability test was conducted for the inverted LacY. In order to screen detergents for their ability to stabilize the inverted LacY topology, we employed a fluorescent thermal denaturation assay using the thiol-specific dye N-[4-(7-diethylamino-4-methyl-3-coumarinyl)phenyl]maleimide (CPM^52^). The stability of LacY purified from PE-lacking cells and solubilized in various detergents was tested using a temperature gradient (Fig. 1B) and as a function of time at 45 °C (Fig. 1C). The results clearly indicate that maltoside detergents (DDM and decyl maltoside (DM)) provide higher stability to the inverted LacY than all other detergent types tested, with DDM showing the highest stability. These results are consistent with the inverted LacY maintaining most of WT LacY TMD arrangement and architecture. However, the inverted LacY is significantly less stable than WT LacY (Fig. 1D), indicating that the changes in inverted LacY’s structural organization have significant effects on its intrinsic conformational stability, possibly extending to substantial impediments for crystallization.

### DDM-solubilized LacY purified from PE-lacking *E. coli* cells maintains its inverted topology, mimicking its *in vivo* arrangement

**Figure 2:**
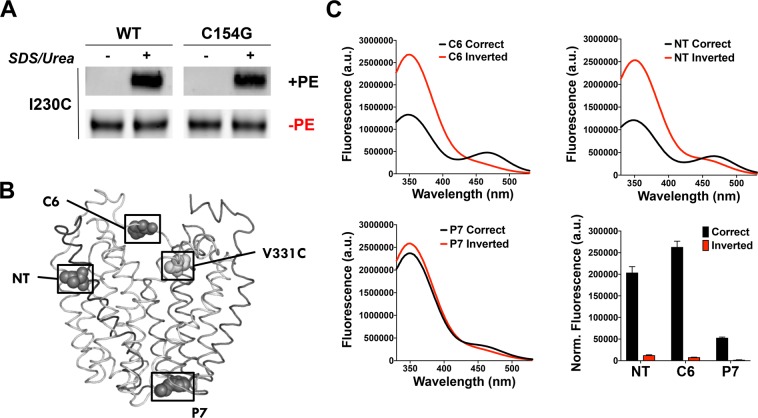
LacY isolated from PE-lacking cells maintains its inverted topology when solubilized in DDM. (**A**) Determination of TMD VII exposure to the aqueous environment using the SCAM^TMD^ assay. Cysteine-less LacY with a single-cysteine replacement in TMD VII (I230C) was purified from PE-containing (+PE) and PE-lacking (−PE) cells and solubilized in DDM and treated with MPB. Samples were subjected to SCAM^TMD^ as described in Methods. Representative results (cropped western blots) are shown for the TMD VII domain in WT LacY, with or without the C154G mutation. (**B-C**) Detection of LacY topology by Trp-IAEDANS FRET. (**B**) Side view of LacY (Protein Data Bank ID code 2CFQ). Diagnostic Trp replacements introduced individually in EMDs NT, C6, and P7 are shown with the indicated positions of Cα atoms of Trp (dark grey spheres). The IAEDANS label is at V331C (light grey sphere). (**C**) Fluorescence of LacY containing a diagnostic Trp replacement in either EMD C6, NT or P7 observed with labeled H205W/V331C, L14W/V331C or F250W/V331C mutant, respectively. a.u., arbitrary units. Spectra were recorded with excitation at 295 nm for 1 μM protein in 50 mM Tris-HCl (pH 7.5), 100 mM NaCl, 0.05% DDM. The bar graph shows the normalized fluorescence intensities at 480 nm, corresponding to the FRET peak, for the three EMDs assessed. The graph represents the average of three different experiments, with error bars indicating SD.

Next, we determined the topology of inverted LacY when solubilized in detergent micelles. A large body of evidence has demonstrated that in the absence of PE, LacY presents with an inverted topology *in vivo* and *in vitro* when reconstituted in proteoliposomes^29,36,38–40^. However, the inverted topology was never established for LacY solubilized and purified from PE-lacking cells using DDM. To do so, we first used a single-cysteine mutant of LacY (in an otherwise cysteine-less protein) with the cysteine strategically located in TMD VII (I230C) in order to test the solvent exposure of TMD VII (Fig. 1A). Using the substituted cysteine accessibility method as applied to TMD determination (SCAM^TMD^)^43^, we determined that I230C, located in TMD VII, is exposed to the aqueous solvent in LacY purified from PE-lacking cells while it is protected in LacY purified from PE-containing cells (Fig. 2A). Additionally, we demonstrated that the C154G mutation (which increases the stability of WT LacY^42^) does not impact TMD VII exposure to the solvent, prompting us to test further if this mutation also provides inverted LacY with improved conformational stability.

We also tested the topology of additional EMDs in the inverted LacY solubilized in DDM using Förster resonance energy transfer (FRET). We previously developed a FRET-based assay^41^ to monitor EMD flipping of LacY in proteoliposomes induced by a post-reconstitution change in lipid composition. Trp residues were introduced in EMD NT, C6 or P7 at positions 14, 205, or 250, respectively. V331C was labeled by 5-(2-Iodoacetyl)-amino-ethyl-amino-naphthalene-1-sulfonic acid (IAEDANS) in the C-terminal TMD bundle (Fig. 2B), whose topology is insensitive to the lipid environment. In the case of NT and C6 EMDs, high FRET indicates native orientation while low FRET indicates the inverted orientation. We measured FRET to assess steady-state topologies of LacY purified from PE-containing (correct topology) and PE-lacking (inverted topology) cells (Fig. 2C) and validated that LacY purified from PE-lacking cells after solubilization in DDM exhibits an inverted topology where NT, C6 and P7 EMDs are all located on the same side of the micelle plane. However, LacY purified from PE-containing cells (correct topology) has NT and C6 EMDs located on the opposite side from P7 EMD. The difference in fluorescence emission for the P7 construct is due to conformational differences between WT and inverted LacY as previously demonstrated by lack of recognition of the inverted LacY by a monoclonal antibody that recognizes an epitope in EMD P7^40^. Altogether, these results allowed us to validate the postulated topologies presented in Fig. 1A.

### Increasing the stability of the inverted LacY using the C154G mutan*t*

**Figure 3:**
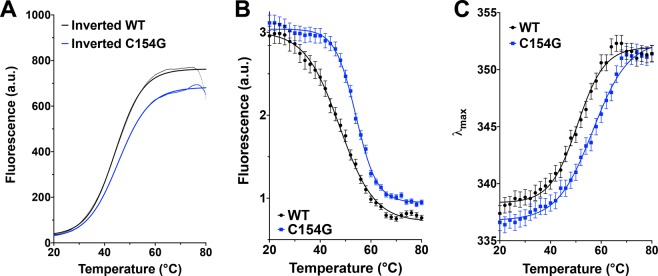
The C154G mutation improves the stability of the inverted LacY in DDM micelles. (**A**) Representative melting curves using the CPM dye binding assay for WT and C154G LacY isolated from PE-lacking cells. LacY was diluted to 26 μg/mL in 50 mM Tris-HCl (pH 7.5), 100 mM NaCl containing 0.05% DDM. (**B**) Intrinsic Trp fluorescence intensity and (**C**) changes in the maximum emission wavelength (λ_max_) of the fluorescence emission spectrum as a function of the temperature for WT and C154G LacY isolated from PE-lacking cells. Protein were diluted to 10 μM in 50 mM Tris-HCl (pH 7.5), 100 mM NaCl containing 0.05% DDM. Solid lines represent log(agonist) *vs*. response (Variable slope) fits of the data. In all cases the experiments were repeated three times and the data represent mean values ± SD.

To further test the stability of the DDM-solubilized inverted LacY, we monitored the thermal denaturation of several LacY templates using both the CPM dye binding assay and intrinsic Trp fluorescent. We first determined that the C154G mutation (which increases WT LacY stability^42^ and Supplemental Fig. 1) does increase the stability of DDM-solubilized inverted LacY, as evidenced by the shift towards higher denaturation temperature (Tm = 45.38 °C for C154G *vs*. Tm = 44.17 °C for WT, as determined by log(agonist) *vs*. response fit of the data) using the CPM dye binding assay (Fig. 3A and Supplemental Fig. 1). In order to validate the findings obtained using the CPM dye binding assay, we also tested the stability of DDM-solubilized LacY by following the intrinsic Trp fluorescence during thermal denaturation. The various LacY mutants used in this study were designed in a LacY template containing all six native Trp residues, which are all located in transmembrane helices (TMD I, II, V, VI, and VII). When located in a hydrophobic environment, Trp residues have a high quantum yield and therefore high fluorescence intensity, while, when exposed to a hydrophilic environment, their quantum yield decreases leading to low fluorescence intensity. As expected, the thermal denaturation of inverted LacY solubilized in DDM followed by Trp fluorescence is observed through a progressive decrease in fluorescence intensity at 350 nm (Fig. 3B, black trace). We also confirm that the C154G mutation increases the stability of inverted LacY, as evidenced by the shift in the denaturation curve towards higher denaturation temperatures (Fig. 3B, blue trace, Tm = 54.09 °C for C154G *vs*. Tm = 47.67 °C for WT, as determined by log(agonist) *vs*. response fit of the data). Additionally, the maximum emission intensity (λ_max_) of a Trp residue excited at 292 nm reflects the polarity of its environment. In a hydrophilic environment, the λ_max_ can reach 355 nm whereas in an apolar environment, the λ_max_ can shift to 320–330 nm. Upon thermal denaturation, the λ_max_ of DDM-solubilized LacY shifts from ~336 nm to ~352 nm (Fig. 3C), indicative of the progressive unfolding of the protein. When the C154G mutation is present, this λ_max_ shift occurs at higher temperatures (Tm = 56.68 °C for C154G vs. Tm = 50.61 °C for WT, as determined by log(agonist) *vs*. response fit of the data), validating that this mutation helps stabilizing the inverted DDM-solubilized LacY.

This side-by-side comparison allowed us to validate the accuracy and validity of the CPM dye binding assay. Since the C154G does not impact TMD VII exposure to the solvent (Fig. 2A) and provides inverted LacY with improved stability, we can conclude that this mutation should be employed in further crystallization studies.

### Inverted LacY exhibits a decreased but significant sugar binding affinity despite alterations in its TMD arrangement

**Figure 4:**
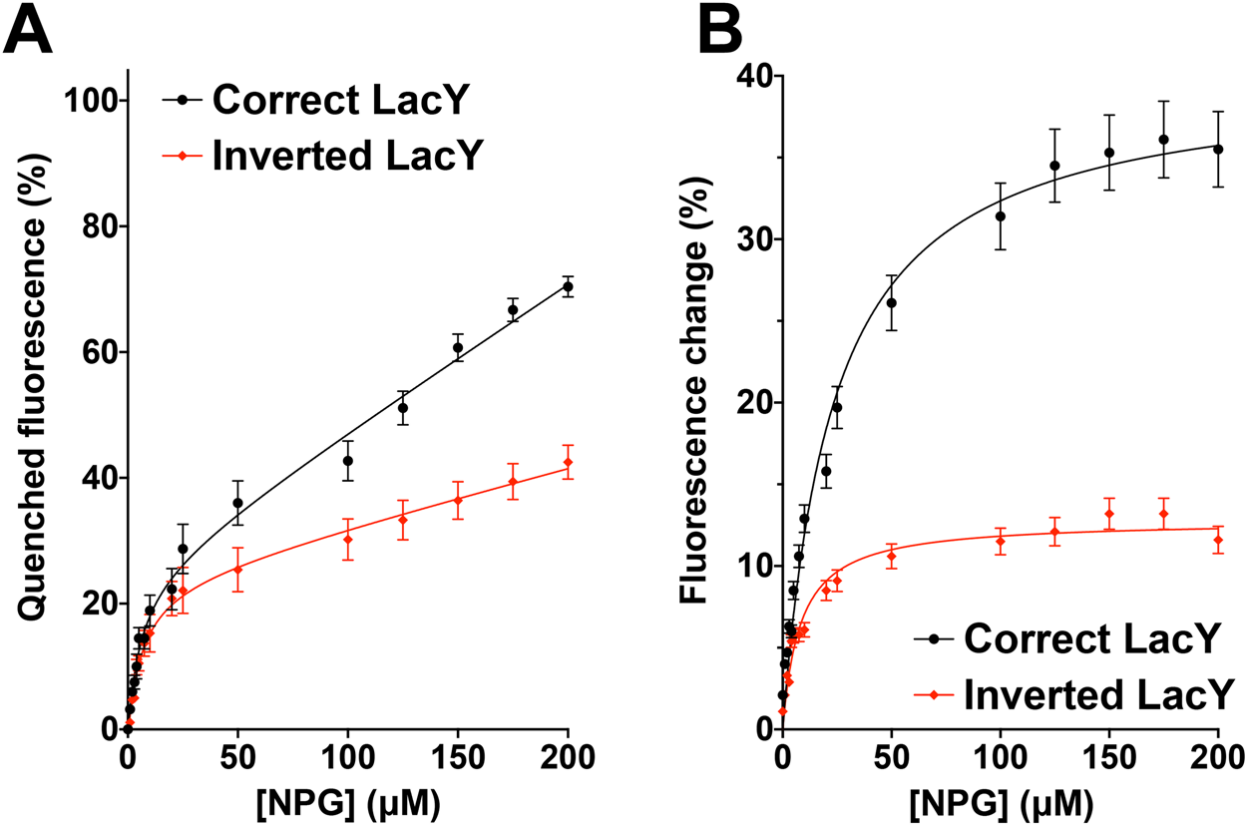
Binding of α-NPG to C154G LacY as detected by Trp → α-NPG FRET. Measurements were carried out in 50 mM Tris-HCl (pH 7.5), 100 mM NaCl containing 0.05% DDM at a protein concentration of 5 μM; excitation was at 295 nm. (**A**) Quenching of Trp fluorescence at different concentrations of α-NPG. (**B**) Affinity of C154G LacY for α-NPG measured by displacement with TDG. The relative fluorescence change after addition of 10 mM TDG is plotted as a function of α-NPG concentration. Fluorescence after TDG addition was taken as 100% at each α-NPG concentration. Solid lines represent Receptor binding – Saturation (One site - Total binding) (**A**) and Michaelis-Menten (**B**) fits of the data. In all cases, the experiments were repeated four times, and the data represent mean values ± SD.

We previously demonstrated that LacY does not undergo major unfolding/refolding events during lipid-dependent topological switching, as evidenced by the lack of significant changes in the intrinsic fluorescence of the six native Trp residues^41^. This observation prompted us to hypothesize that the N-terminal bundle made of 6 TMDs may have conserved a somewhat compact arrangement in the membrane environment. In order to assess the TMD bundle organization, we tested the ability of DDM-solubilized LacY to bind the relatively high-affinity lactose analogues β-D-galactopyranosyl-1-thio-β-D-galactopyranoside (TDG) and 4-nitrophenyl- β-D-galactopyranoside (α-NPG). To do so, we adapted a method developed by the Kaback group using a LacY template carrying a single Trp residue (W151) in the sugar-binding site^53^. This method takes advantage of the specific interaction of an aromatic residue at position 151 (located in TMD V), with the galactopyranosyl ring. Indeed, they demonstrated that formation of a donor-acceptor pair between W151 and α-NPG in the sugar-binding site results in Trp → α-NPG FRET signal, which can be abolished by addition of TDG^54,55^. We conducted fluorescent measurements on DDM-solubilized LacY containing the C154G mutation and all six native Trp residues (including W151) purified from PE-containing (correct topology) and PE-lacking cells (inverted topology). Addition of α-NPG, which is the acceptor in Trp → α-NPG FRET, results in a progressive quenching of W151 as a function of α-NPG concentration (Fig. 4A and Supplemental Fig. 2). The data are best fitted using the Receptor binding-Saturation (One site-Total binding) equation, allowing the extrapolation of the equilibrium dissociation constant (Kd) for NPG: 5.896 μM for the correct LacY *vs*. 6.276 μM for the inverted LacY, indicative of lower α-NPG binding efficiency.

When incubating the LacY/α-NPG complex with 10 mM TDG, a dequenching of W151 can be observed in both correctly oriented and inverted LacY (Fig. 4B). Unlike α-NPG quenching, TDG dequenching curves are best fitted using a Michaelis-Menten equation, allowing for the extrapolation of the Michaelis-Menten constant (Km), which in our case indirectly reflects the binding constant of TDG. The values obtained indicate a higher Km for correct LacY (23.38 μM) compared to inverted LacY (8.129 μM). These findings demonstrate that the correct LacY has a lower affinity for α-NPG in the presence of 10 mM TDG than the inverted LacY, indicating a higher TDG binding affinity in the correct LacY compared to the inverted topology. Altogether, these results indicate that the inverted LacY solubilized in DDM is still able to bind both α-NPG and TDG, but with a lower affinity than the correctly oriented LacY, thus suggesting the maintenance of significant structural organization and interaction among the 11 TMDs. This arrangement leads to the formation of TMD-TMD interactions similar enough to the ones present in WT LacY to support binding of α-NPG and TDG to the inverted LacY, but dissimilar enough that it decreases their relative affinity. This finding hints at the possibility of using LacY’s substrate to improve its stability, as the inverted topology does not totally prevent TDG from binding to LacY. Also, this observation prompts a deeper assessment of the structural arrangement of these TMDs in the inverted topology, with special emphasis on their tilt angle in the membrane and inter-TMD residue interactions.

### The inverted LacY exhibits a decreased helicity content due to the loss of secondary structure in TMD VII

**Figure 5:**
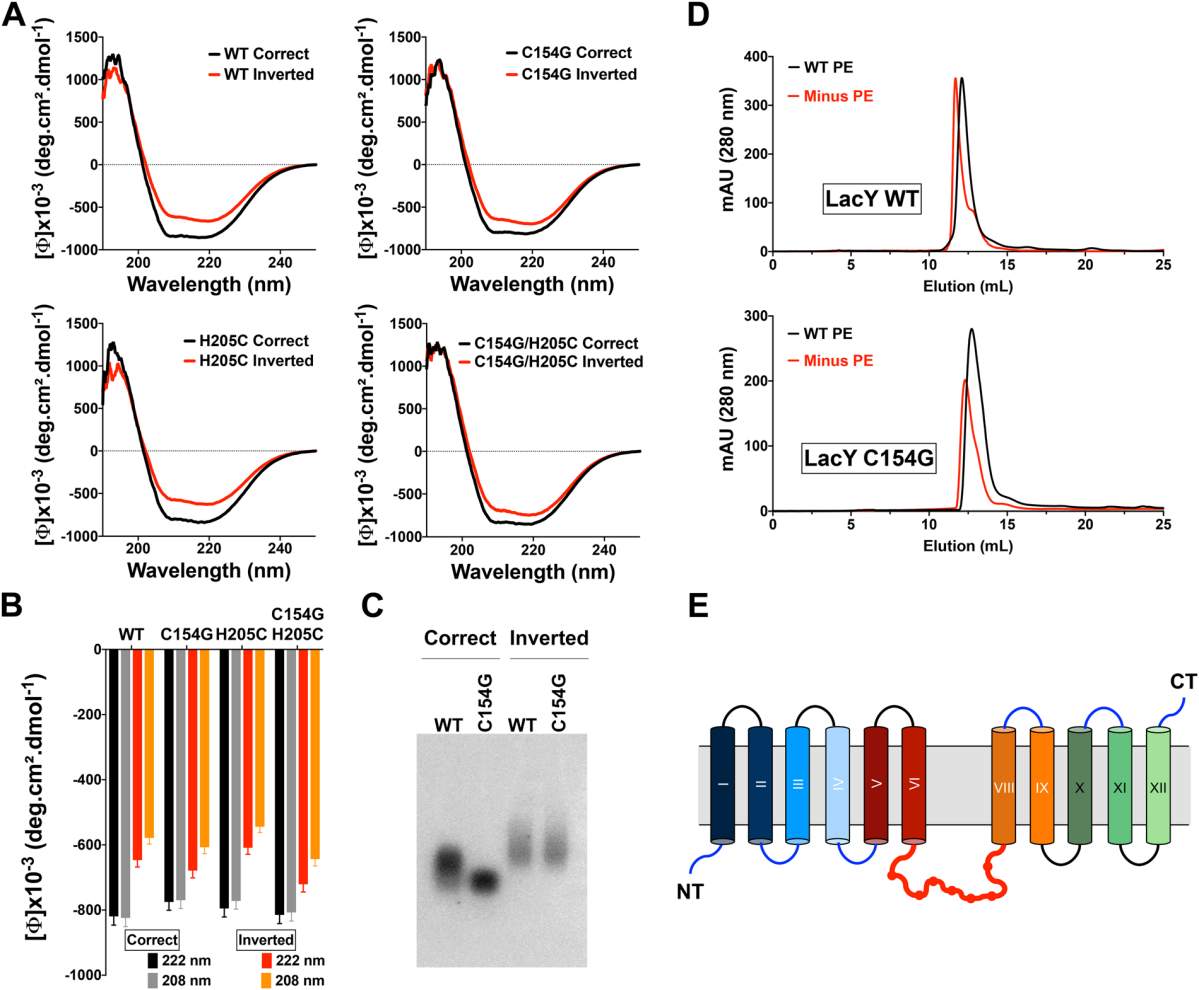
Loss of TMD VII secondary structure arrangement in the inverted LacY solubilized in DDM micelles. (**A**) Far-UV circular dichroism spectra of DDM-solubilized LacY isolated from PE-containing (correct topology, black lines) and PE-lacking (inverted topology, red lines). (**B**) Mean residue ellipticities at 222 and 208 nm for the correct and inverted LacY topologies in WT, C154G, H205C and C154G/H205C templates. (**C**) Non-denaturing agarose gel depicting migration of correctly oriented and inverted WT LacY, with or without the C154G mutation. (**D**) SEC analysis of LacY WT and C154G purified from PE-containing (WT PE) and PE-lacking (Minus PE) cells and solubilized in DDM. (**E**) Topological organization of LacY purified from PE-lacking cells indicating the absence of secondary structure in the now solvent-exposed TMD VII.

The conformation-specific monoclonal antibody 4B1 recognizes native LacY via an epitope in domain P7^56,57^. Previous work from our laboratory demonstrated that the misorientation of the N-terminal six-TMD bundle of LacY in PE-lacking cells disrupts the native structure of EMD P7^30,31^, inferring a loss of specific structural features. While the structural arrangement of correctly oriented LacY has been resolved at high resolution, no assessment of the overall levels of secondary structure has been reported for the inverted LacY. To address this, we characterized the secondary structure of DDM-solubilized LacY purified from PE-containing and PE-lacking cells using circular dichroism. We used three different LacY templates containing the C154G mutation and/or a single cysteine replacement in the EMD C6 (H205C) and compared them to WT LacY. In all cases, DDM-solubilized LacY exhibits typical features found in α-helices-containing proteins with two minima at 208 and 222 nm (Fig. 5A, black traces). Interestingly, all templates purified from PE-lacking cells also exhibit these two minima but with increased value, indicating a decrease in the helicity content of LacY (Fig. 5A, red traces). By monitoring the signal intensity at 208 and 222 nm (Fig. 5B), we estimated that the inverted LacY has a lower total helicity content than the correctly oriented LacY (68–71% in the inverted compared to 84–88% in the correct), independently of the template analyzed. This strongly indicates that the exposure of TMD VII to the aqueous environment is accompanied by a loss of secondary structure.

To further test this, we subjected WT and C154G LacY templates purified from PE-containing and PE-lacking cells to electrophoretic migration in a 1% agarose native gel. Differences of migration were observed between the WT and C154G mutation in the correctly oriented LacY, indicating a more compact/less flexible structure when the C154G mutation is present (Fig. 5C). Decrease in LacY flexibility in the C154G mutant was previously observed in the first published crystal structure of LacY where the C154G mutation resulted in more stable crystals amenable to diffraction at high resolution^42^. The inverted LacY presented with even slower migration than the WT LacY, indicating that the exposure of TMD VII to the solvent increases the overall average radius of the inverted LacY, possibly due to higher flexibility of DDM-solubilized LacY and the unfolding of TMD VII. This increase in flexibility was observed irrespective of the C154G mutation. We further confirmed these changes in protein compactness by submitting the affinity-purified LacYs to size exclusion chromatography (SEC) on Superdex 200. Representative elution profiles are presented in Fig. 5D for LacY WT and C154G purified from PE-containing (WT) and PE-lacking (Minus PE) cells. Lower retention times are observed for LacY purified from PE-lacking cells (11.69 min for LacY WT and 12.31 min for C154G), indicating a “higher molecular weight”, i.e. a higher Stokes radius, compared to LacY purified from PE-containing cells (12.11 min for LacY WT and 12.73 min for C154G). We also confirm that the mutation C154G helps stabilizing LacY and presents with a more compact conformation, compared to the WT template, as indicated by higher retention times (i.e. lower Stokes radius). Additionally, the elution profile of LacY purified from PE-lacking cells indicates larger heterogeneity in the protein with an asymmetrical elution peak. These findings suggest that, despite providing higher stability for the inverted LacY, the C154G mutation does not result in new interactions between TMD V and the now solvent-exposed TMD VII. Altogether, these results suggest that, as a consequence of the exposure and unfolding of TMD VII in the aqueous environment, the inverted LacY is less compact and less structured than the correctly oriented LacY (Fig. 5E).

### Improving the stability of inverted LacY in detergent micelles correlates with crystal formation

Based on the various biophysical characterization of DDM-solubilized LacY in its inverted topology, we reasoned that increasing the stability of the inverted protein in detergent micelles to a level similar to the correctly oriented LacY is a mandatory step towards the generation of stable 2D crystals. To do so, we tested the effects of several additives, as well as pH and the incorporation of phospholipids into the DDM micelles. To test the possible stabilizing effects of lipids supplied to the DDM micelles, we first validated that lipids could be integrated into protein-containing DDM micelles. To do so, we used brominated lipids and measured the fluorescence quenching of LacY’s native Trp residues upon lipid re-supply. The results (Supplemental Fig. 3) indicate that the incubation of DDM-solubilized LacY with DDM micelles containing an increasing content in brominated-phosphatidylcholine (BrPC) results in a progressive decrease in the intrinsic Trp fluorescence of LacY, irrespective of the template used (WT, C154G or H205C/C154G), demonstrating that phospholipids can be transferred to the LacY-containing DDM micelles.

**Figure 6:**
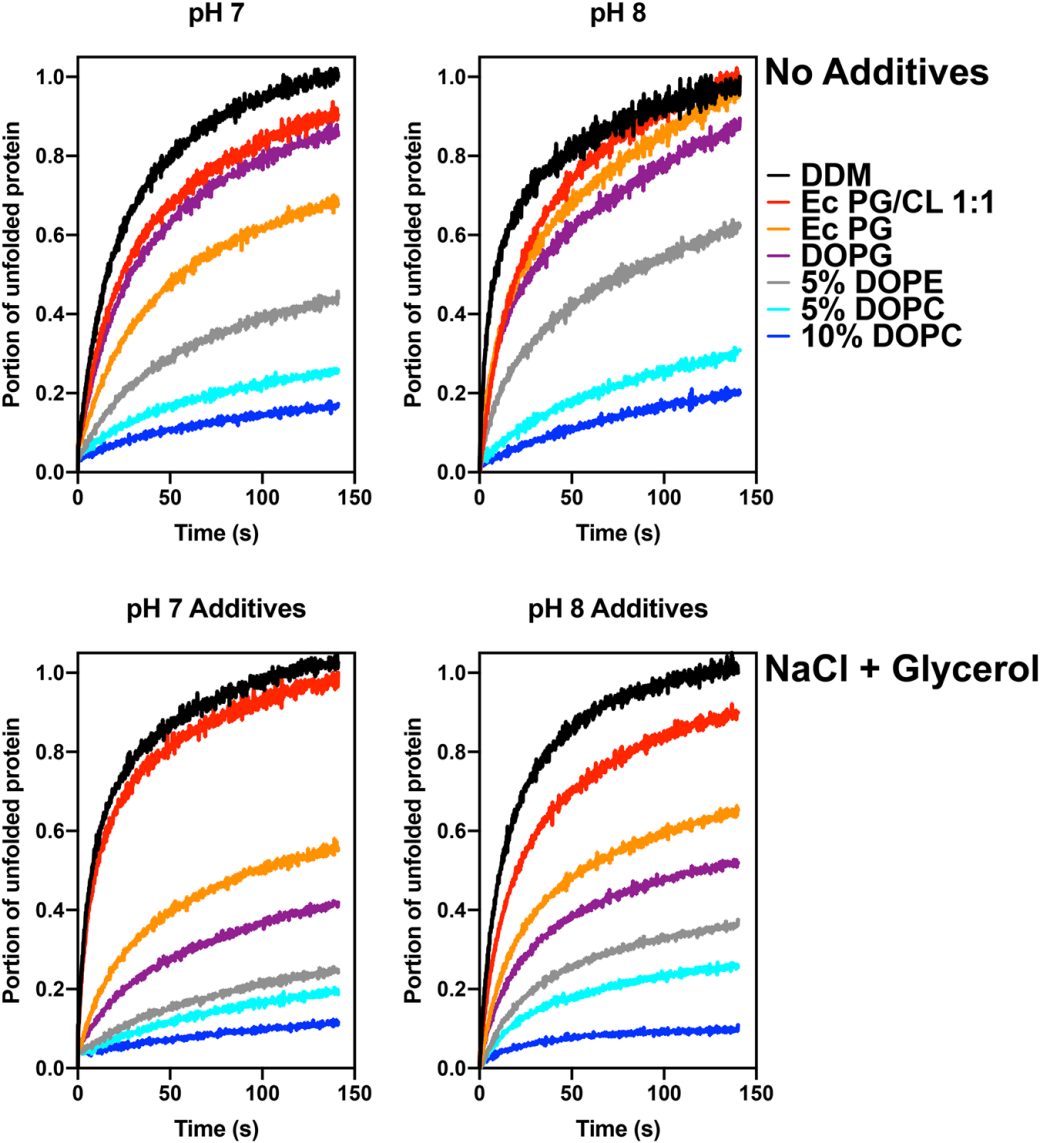
Resupply of zwitterionic phospholipids and additives improve the stability of the inverted LacY in DDM micelles. Representative unfolding curves at 45 °C for 150 min for LacY C154G/H205C purified from PE-lacking cells and solubilized in DDM. LacY was diluted to 26 μg/mL in 50 mM Tris-HCl at pH 7 (left panels) or pH 8 (right panels) and in the presence of 150 mM NaCl (no additives, top panels) or 250 mM NaCl and 10% glycerol (NaCl + Glycerol, right panels). Resupply of phospholipids was conducted prior to thermostability assessment by incubation with DDM-solubilized phospholipids.

The stability of the inverted LacY (C154G/H205C template) in DDM micelles was then monitored using the CPM dye binding assay conducted at 45 °C, over a 3-h time period. The experiments were conducted at two different pHs and in the presence of additives and/or after incubation with DDM-solubilized phospholipids. Several observations can be made from the results presented in Fig. 6. First, the addition of NaCl and glycerol appear to be most beneficial when *E*. *coli* PG, dioleyl-PG (DOPG), DOPE and DOPC are also present. It appears to have only mild to moderate effects when *E*. *coli* CL is present in the micelles. Also, while no major differences are observed between pH 7 and 8, pH 8 provides higher stability to the inverted LacY. Finally, we tested multiple lipid combinations added back to the DDM-solubilized LacY. Given the fact that the inverted LacY was isolated from PE-lacking cells and that the purification protocol utilizes only one chromatographic step, there are still many native lipid molecules bound to LacY, as previously demonstrated^48^. We therefore can expect the micelles to contain several molecules of *E*. *coli* PG and CL, the two major phospholipids remaining in PE-lacking cells.

Several phospholipids were tested for their ability to improve the stability of LacY, including *E*. *coli* PG/CL mixtures, *E*. *coli* PG, DOPG, dioleyl-PE (DOPE) and dioleyl-PC (DOPC). For the various combinations tested, validation that phospholipid resupply did not change the topological arrangement of LacY was verified using the I230C template by conducting SCAM^TMD^ analysis (Supplemental Fig. 4). While the lack of an effect on topology from PG or PG/CL resupply was expected, we observed that resupply of PE or PC did not alter exposure of TMD VII to the aqueous environment when limited to 10% or less, while mixed topology features were observed when levels were raised to 20%, mimicking the results previously obtained in proteoliposomes^40^.

The results presented in Fig. 6 indicate that decreasing the CL content by addition of either *E*. *coli* PG/CL 2:1, *E*. *coli* PG or DOPG leads to improvements in protein stability, suggesting that CL participates in destabilizing the protein. Additionally, homogenization of the fatty acid composition of PG (from a mixture of 14:0, 15:0, 16:0, 16:1, cyclo17:0, 18:0, 18:1 and cyclo19:0 in *E*. *coli* PG extract^58^ to 18:1 only in DOPG) stabilizes the protein further, indicating a direct interaction between LacY and PG. Finally, addition of up to 10% of DOPC significantly increases the stability of the inverted LacY, while addition of DOPE leads to similar, although less efficient, results.

**Figure 7:**
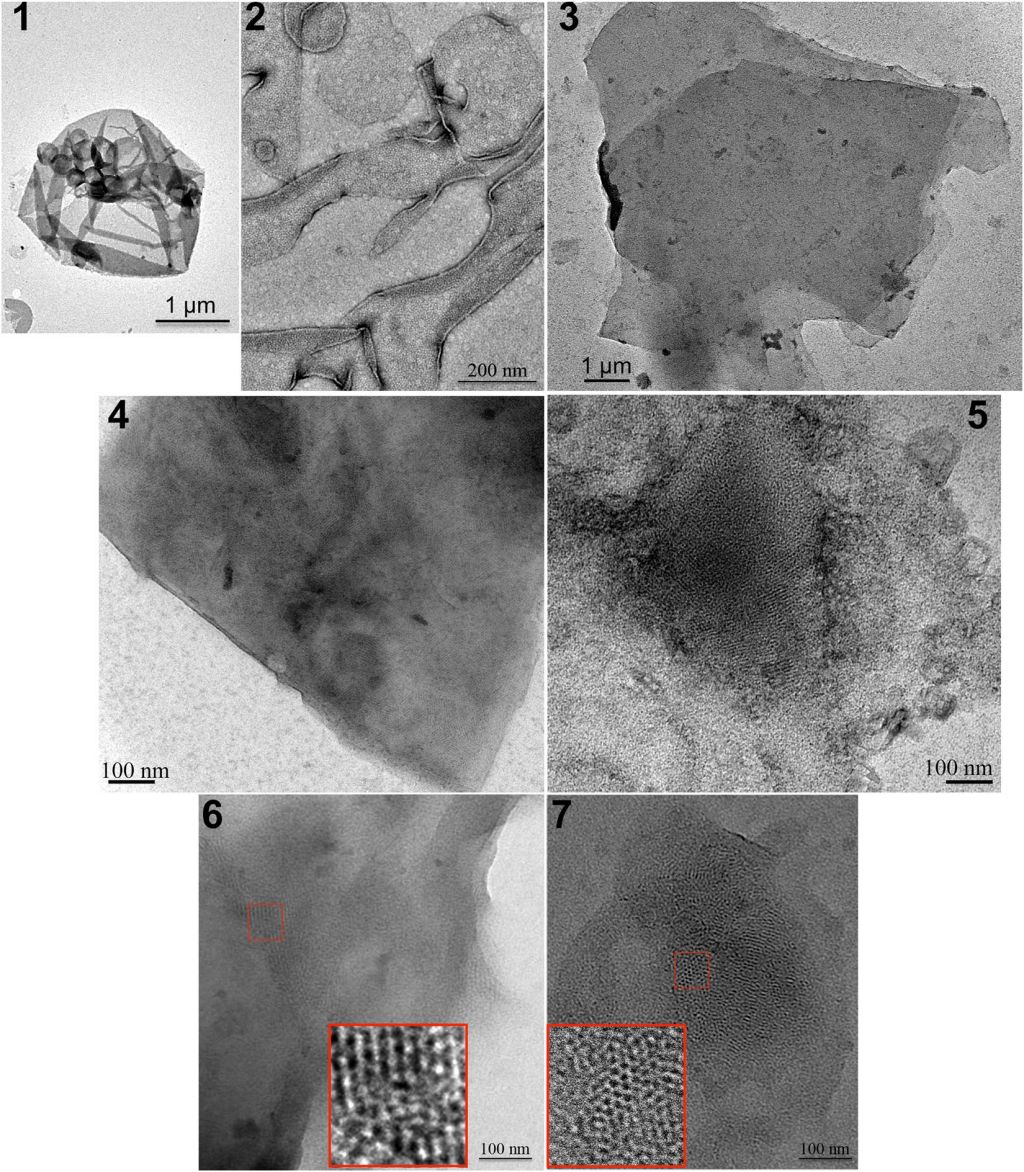
Electron microscopy of representative crystallization outcomes of the inverted LacY C154G/H205C. Overview of a negatively stained sample depicting the presence of protein reconstitution in densely packed proteoliposomes (panel 1), membrane sheets (panel 2), membrane stacks (panel 3), and patchy crystals (panels 4 to 7). The inverted LacY is visible in patchy crystals areas imaged at higher magnification (panels 6 and 7). Scale bars represent 1 μm in (1) and (3), 200 nm in (2), and 100 nm in (4 to 7).

Next, we conducted 2D crystallization trials using the inverted LacY carrying the stabilizing mutation C154G and a single cysteine in EMD C6 (H205C) purified using DDM from a PE-lacking strain. The various conditions tested (such as lipids used in the tertiary mixture, protein concentration, lipid-to-protein ratio, additives) as well as the outcome are documented in Supplemental Table 1. We also verified that the reconstitution of LacY in phospholipid membranes at low lipid-to-protein ratios resulted in the maintenance of its inverted topology by conducting SCAM^TMD^ analysis (Supplemental Fig. 5). Representative results of crystallization outcomes are presented in Fig. 7, depicting the presence of protein reconstitution in densely packed proteoliposomes (Fig. 7, panel 1), membrane sheets (Fig. 7, panel 2), membrane stacks (Fig. 7, panel 3), and patchy crystals (Fig. 7, panels 4 to 7). More detailed representative images of the types of crystals obtained are presented in Fig. 7, panels 6 and 7, where the dense arrangement of LacY in patchy crystals is visible.

The set of conditions leading to the generation of crystals, compiled in Table 1, indicates that (i) divalent cations such as MgCl_2_ and CaCl_2_ help stabilizing membranes containing CL. This stabilization is further improved by other additives (glycerol) when CL is present at higher levels, but leads to a high rate of aggregation and stack formation, hindering data collection. The best results were obtained in the presence of the DOPG:DOPC 9:1 mixture and 200 mM MgCl_2_, requiring low protein concentration and low lipid to protein ratio (LPR). Some promising results were also obtained with DOPG in the presence of CoCl_2_ and NaCl where aggregation was very low and large vesicle sheets containing patchy crystals formed in less than 5 days, although higher LacY concentration and/or addition of substrate were necessary. Altogether these preliminary crystallization results indicate that efforts aimed at stabilizing inverted LacY in DDM micelles using resupply of lipids, substrate binding and membrane stabilization with divalent cations and additives help improve crystallization outcome while maintaining LacY’s inverted topology.

**Table 1:** Two-dimensional crystallization conditions for the inverted LacY leading to crystal formation.

Lipids	[LacY]^a^	LPR	Divalent cations^b^	Monovalent ions	Other	Ligand^c^	T^d^	pH	Results
DOPG/DOPC 9:1	1	0.75	200 mM MgCl_2_	—	—	None	4 °C	5	**Patchy Crystals** Large vesicles, Sheets
DOPG/DOPC 9:1	1	0.75	200 mM MgCl_2_	—	—	None	4 °C	8	**Patchy Crystals** Large vesicles, Sheets
DOPG	1	1	CoCl_2_	50 mM NaCl	—	TDG	RT	8	**Patchy crystal**
DOPG	2.5	1	None	50 mM NaCl	—	None	RT	8	**Patchy Crystals** Large vesicles, Sheets
DOPG	2.5	1	CoCl_2_	50 mM NaCl	—	TDG	RT	8	**Patchy crystals** Vesicles, Sheets
DOPG/CL(18:1) 9:1	1	0.75	200 mM MgCl_2_ CaCl_2_	—	—	None	RT	8	**Small Patchy Crystals** Sheets, Stacks
DOPG/CL(18:1) 9:1	1	0.75	200 mM MgCl_2_ CaCl_2_	50 mM LiCl	—	None	RT	8	**Small Patchy Crystals** Large Sheets, Stacks
DOPG/CL(18:1) 9:1	1	0.75	200 mM MgCl_2_ CaCl_2_	—	—	None	37 °C	8	**Patchy Crystals** Large Sheets, Stacks
DOPG/CL(18:1) 9:1	1	0.75	200 mM MgCl_2_ CaCl_2_	50 mM KCl	—	None	37 °C	8	**Patchy Crystals** Large Sheets, Stacks
PG/CL 1:1	0.5	0.75	200 mM MgCl_2_	—	—	None	RT	8	**Patchy Crystals** *Aggregation*, Vesicles, Stacks
PG/CL 1:1	1	0.2	50 mM MgCl_2_	—	20% glycerol	None	RT	8	**Patchy Crystals** *Aggregation*, Vesicles
PG/CL 1:1	1	0.5	50 mM MgCl_2_	—	20% glycerol	None	RT	8	**Patchy Crystals** *Aggregation*, Vesicles
PG/CL 2:1	1	0.2	50 mM MgCl_2_	—	20% glycerol	None	RT	8	**Patchy Crystals** *Aggregation*, Vesicles, Stacks
PG/CL 2:1	1	0.5	50 mM MgCl_2_	—	20% glycerol	None	RT	8	**Patchy Crystals** *Aggregation*, Vesicles, Stacks
PG/CL 2:1	1	1	200 mM MgCl_2_	—	—	None	RT	8	**Patchy Crystals** *Aggregation*, Vesicles, Stacks

^a^LacY concentration in mg/mL.

^b^CoCl_2_ and CaCl_2_ at 10 mM.

^c^TDG at 10 mM.

^d^Temperature at which the dialysis is conducted.

## Discussion

A large body of evidence supports a close relationship between functional and gross structural details observed for WT LacY in its native lipid environment and LacY purified using detergents^42,44,49^. Therefore, conclusions based on the high-resolution crystal structure of purified WT LacY can be extended to the protein in its native environment. Structural studies on LacY and many membrane proteins support the Positive Inside Rule^59^, which is based on the statistical and experimentally determined observation that the majority of membrane protein EMDs facing the cytoplasm have a net positive charge while those facing the trans side are negative or neutral thus defining the orientation of TMDs residing in the lipid bilayer. Extensive studies on the effect of lipid environment on the structure and function of LacY, as well as several other secondary transporters, in cell membranes and proteoliposomes has established lipid-protein interactions as a defining parameter in determining membrane protein structure and function^60–62^. These studies have extended the Positive Inside Rule to the Charge Balance Rule, which incorporates the effect of charge interactions between membrane lipid head groups and EMDs of proteins in establishing TMD orientation in the lipid bilayer^36,61^.

The presence of net neutral lipids such as PE and PC suppresses the effect of negative residues within EMDs as protein membrane translocation signals thereby strengthening the cytoplasmic retention signal of positive residues. This effect explains the cytoplasmic location of EMDs having an apparent net negative charge. The ratio of two membrane protein topological conformers can be adjusted as a continuum between two extremes by altering the net charge of EMDs or changing the ratio of net neutral to anionic phospholipids^34,40^. Thus, the Charge Balance Rule provides the basis for proteins that display multiple topologies or change their topological organization in response to changes in their lipid environment or post-translational modification^60^. In order to further our understanding of how lipid-protein interactions govern protein structure, high-resolution structures of membrane proteins as a function of lipid environment must be obtained. However, solubilized proteins must first be verified to reflect the structure and function of the protein in the cell membrane.

Studies reported herein establish that the lipid-dependent structure/function of LacY can be recapitulated and analyzed in phospholipid-containing detergent micelles. Several detergents were screened to identify optimal solubilization conditions for stability. Similar to results obtained for the correctly oriented LacY, DDM is the detergent of choice for solubilizing and stabilizing the inverted LacY. The results also indicate a correlation between increasing detergent micellar size and LacY stability, hinting at the necessity to retain sufficient phospholipids in close proximity of the TMDs. LacY purified from PE-lacking cells exhibits an inverted topology in DDM micelles, while maintaining significant level of substrate binding ability. The results point toward the conservation of an interaction network between TMDs still embedded in the hydrophobic region, despite the dramatic rearrangement of the N-terminal 6-TMD bundle. This observation is consistent with *in vivo* data indicating that whole cells or proteoliposomes containing primarily PG/CL and lacking net neutral lipids exhibit energy-independent facilitated but not energy-dependent active transport^38,63^. This suggests maintenance of a certain degree of compactness and interactions between the various TMDs still embedded in the membrane. The C-terminal 5-TMD bundle of the inverted LacY does not undergo any topological rearrangement and should therefore be able to maintain its inter-TMD interactions. On the other hand, the inverted N-terminal 6-TMD bundle reorients itself in the membrane in the inverted LacY but appears to do so as a single-unit^41^, implying the possibility of maintenance and/or re-establishment of its inter-TMD interactions. Further, evidence of substrate binding ability, although partial, leads us to postulate that the two helical bundles engage in specific TMD-TMD interactions, despite the absence of TMD VII inside the hydrophobic core of the micelles.

In inverted LacY, solvent-exposed TMD VII does not present with secondary structure, yielding a large solvent-exposed domain made of EMD C6, former TMD VII and EMD P7. This domain appears largely unstructured, presenting a possible hindrance to crystallization efforts. Previous 2D crystallization attempts were made using a chimeric version of LacY (with correct topology) containing Cytb_562_ (nicknamed the “red permease”) inserted in EMD C6^64^. The rationale was based on the ease to follow the fate of the protein during purification and crystallization, as well as providing increased stability and polar surface area in the EMD C6 region. This early work by Engel and colleagues already highlighted the importance of maintaining sufficient levels of annular lipids for crystal growth and pointed out the high level of flexibility observed in the EMD C6 region. While our initial screen shows promise regarding crystal formation by focusing on TMDs and lipid-protein interactions, further efforts should be pursued aiming at (i) stabilizing this long unstructured domain and (ii) preventing its interactions with other units of LacY inside the crystal lattice. Indeed, one of the main issues encountered while imaging was the large presence of membranes sheets stacked on top of each other. Strategies involving labeling of this domain with large stabilizing compounds (such as biotin or monoclonal antibodies) or 2D crystallization on a lipid monolayer^65^ may help improve the quality of the crystals obtained.

Several protein structural features and conditions increase the stability of inverted LacY while maintaining its inverted topology: (i) presence of the C154G mutation, (ii) supplementation with 5–10% DOPC, (iii) use of phospholipids with a homogenous fatty acid composition and (iv) additives such as glycerol, NaCl, divalent cations. Improved stability of LacY correlates with crystal formation. Biochemical characterization of native LacY by the Kaback laboratory provided a gross organization of TMs and extramembrane domains^44^, which were subsequently verified by high-resolution crystallographic techniques^42,49^. We expect that obtaining high-resolution structural information on LacY isolated from cells with different lipid compositions will provide new and unique insight into the molecular details of lipid-dependent topogenesis. The 2D crystallization process requires the incorporation of the target membrane protein into a defined lipid bilayer while most 3D crystallization techniques maintain the target membrane protein in non-physiological detergent-rich environment^9^, making 2D crystallization the method of choice for lipid-dependent structural characterization of two oppositely oriented conformers of LacY.

More detailed structural information is needed to understand the organization of the region C6-TMD VII-P7 when PE is lacking, as well as the TMD I-P1-TMD II-C2 domain after introduction of PE post-assembly of LacY^36^. In the latter case, LacY regains uphill transport function yet there are residues within the NT domain that are required for activity in spite of apparently being mis-organized. How are these residues now organized in this structure? There are subtle differences in the organization of the periplasmic domains when LacY is assembled in monoglucosyldiacylglycerol- (MGlcDAG) containing membranes versus native membranes^33,66,67^. Similarly, cysteine replacements within periplasmic domains P3 and P5 were only accessible to MPB labeling at higher pH when LacY is expressed in *E*. *coli* cells where PE is replaced by PC, indicating changes in the local secondary structure/environment of these cysteines compared to LacY expressed in WT *E*. *coli* cells^28^. Perturbation of the structure of EMD P7 also affects function^68^.

Further studies systematically addressing how the presence of foreign lipids affect high-resolution structural determination, which complement our current studies on lipid-dependent topogenesis, are needed. Many membrane protein crystals are still grown in the presence of synthetic PC ignoring the fact that synthetic PC can unfold and disable functionally important elements of secondary structure during refolding of proteins for which PC is not native^30,31^. The current extensive database on LacY structure, function and organization as a function of lipid environment makes LacY an excellent protein to carry out more extensive high-resolution structural determination, while similar studies can be extended to other proteins whose organization is lipid-sensitive.

## Methods

### Reagents

All lipids were purchased from Avanti Polar Lipids: L-α-phosphatidylethanolamine (PE) from *E*. *coli*, L-α-phosphatidylglycerol (PG) from *E*. *coli*, cardiolipin (CL) from *E*. *coli*, 1,2-dioleoyl-*sn*-glycero-3-phosphoethanolamine (DOPE), 1,2-dioleoyl-*sn*-glycero-3-phospho-(1′-rac-glycerol) (DOPG), 1,2-dioleoyl-*sn*-glycero-3-phosphocholine (DOPC), 1-palmitoyl-2-(9,10-dibromo)stearoyl-*sn*-glycero-3-phosphocholine (16:0–18:0(9-10Br)PC), and 1′,3′-bis[1,2-dioleoyl-*sn*-glycero-3-phospho]-glycerol (18:1 CL). All detergents were purchased from Anatrace: 3-[(3-Cholamidopropyl)dimethylammonio]-1-propanesulfonate (CHAPS), octylglucoside (OG), polyoxyethylene(9)dodecyl ether (C12E9), n-Decyl-α-D-Maltoside (DM), n-Dodecyl-α-D-Maltoside (DDM), lauryldimethylamine-N-Oxide (LDAO). The Imperial Protein Stain solution and micro-bicinchoninic acid assay (BCA) were purchased from Thermo Fisher. The QuikChange Lightning site-directed mutagenesis kit was purchased from Agilent Technologies. 1,5-((((2-iodoacetyl)amino)ethyl)amino)naphthalene-1-sulfonic acid (IAEDANS) and 3-(N-maleimidopropionyl) biocytin (MPB) were purchased from Molecular Probes. 4-Nitrophenyl- β-D-galactopyranoside (NPG) and N-[4-(7-diethylamino-4-methyl-3-coumarinyl)phenyl]maleimide (CPM) were purchased from Sigma Aldrich. β-D-Galactopyranosyl 1-thio-β-D-galactopyranoside (TDG) was obtained from Cayman Chemicals.

### Bacterial strains, plasmids, growth condition, and lacy purification

Strain AL95 [*pss93::kan*^*R*^ *lacY::Tn9* (kanamycin resistance gene inserted into gene encoding phosphatidylserine synthase and tetracycline resistance gene inserted into gene encoding lactose permease (LacY), respectively)] with (wild-type lipid composition) or without (lacking PE and containing mostly PG and CL) plasmid pDD72GM [*pssA*^+^ *gen*^*R*^ (gene encoding phosphatidylserine synthase and gene encoding gentamycin resistance, respectively) on plasmid pSC101 with a temperature-sensitive replicon]^29^ were used as the host for expression of genes encoding LacY derivatives carried on the following plasmids. Plasmid pT7-5/C-less LacY (amp^R^, ColE1 replicon), provided by H. R. Kaback and encoding LacY in which Cys residues are replaced by Ser (LacY*), was used to construct derivative of LacY. Plasmid pT7-5/C-less LacY/I230C^36^ was used for monitoring exposure of TMD VII to the solvent by substituted cysteine accessibility method as applied to TMD determination (SCAM^TMD^). The QuickChange Lightning site-directed mutagenesis kit was used to construct the plasmid derivative expressing LacY* I230C/C154G and LacY* H205C/C154G. Plasmids pT7-5/C-less LacY/V331C, where Trp replacements were made in EMDs NT, C6 and P7 at position 14, 205 and 250, respectively^41^, were used for monitoring of TMD flipping within LacY by FRET. The templates encoding for LacY WT, LacY C154G and LacY* H205C have been previously described^36,39,42^.

LacY was engineered with a His_6_-Tag at the C-terminus to facilitate purification and was expressed under control of LacY operator/promoter by growth of cells in the presence of 1 mM isopropyl-β-thiogalactopyranoside (IPTG). Cells were grown in LB-rich medium containing ampicillin (100 μg/mL) to an OD_600_ of 0.6, induced by addition of IPTG, and grown until cell arrest occurred. Purification of LacY was carried out at 4 °C or on ice as previously described^39^. Protein content during purification and the concentration of LacY in proteoliposomes were determined by the micro-BCA protein assay, according to the manufacturer’s instructions.

### IAEDANS labeling

Trp replacement derivatives of LacY/V331C were purified from PE-containing and PE-lacking cells. Labeling of LacY with IAEDANS was carried out as previously described^41^. The labeling ratio was quantified by comparing the absorption spectrum of labeled protein with unlabeled protein using extinction coefficients of ε_334_ = 5,700 M^−1^.cm^−1^ for IAEDANS. Typical labeling efficiency was above 85%.

### Preparation of proteoliposomes

Proteoliposomes were formed by reconstitution of protein into small unilamellar liposomes of various lipid compositions as previously described^39^ by incorporation of LacY into preformed liposomes.

### Chemical protein topology mapping

Topological determination of TMDs (SCAM^TMD^) is based on the controlled membrane permeability of the thiol-specific reagent MPB and its reactivity with diagnostic cysteine residues in LacY as previously described^39^, with slight modifications. In brief, 100% cysteine labeling was obtained after solubilizing/denaturing LacY-containing micelles with addition of 2% SDS and 2 M urea before conducting cysteine labeling. Using LacY with a single cysteine engineered in TMD VII (I230C), we monitored the accessibility of TMD VII to the solvent (reflecting the inverted LacY topology).

### Topological assessment by FRET

The fluorescence measurements were conducted using a QuantaMaster model QM3-SS (Photon Technology International), a cuvette-based fluorescence spectrometer. Using a Peltier TE temperature controller, the sample was held at a constant 20 °C. All measurements were conducted in degassed 50 mM Tris-HCl (pH 7.5)/100 mM NaCl/0.05% DDM. Data were collected and analyzed using Felix 32 software. The DDM-solubilized LacY (containing 1 µM of IAEDANS–labeled LacY) was first equilibrated at 20 °C, then FRET was monitored at an excitation wavelength of 295 nm (for Trp) and at an emission wavelength of 475 nm (for IAEDANS), both with a 1-nm band pass. We used the FRET values observed for WT LacY purified from PE-containing *E*. *coli* (F_WT_) to normalize the FRET values (F_Meas_), using equation 1.

1 $${{\rm{F}}}_{{\rm{norm}}}={{\rm{F}}}_{{\rm{Meas}}}/{{\rm{F}}}_{{\rm{WT}}}$$

### Fluorescence extinction of LacY in the presence of brominated phospholipids

The fluorescence measurements were conducted using a QuantaMaster model QM3-SS (Photon Technology International), a cuvette-based fluorescence spectrometer. Data were collected and analyzed using Felix 32 software. All measurements were conducted in degassed 50 mM Tris-HCl (pH 7.5)/100 mM NaCl/0.05% DDM. Using a Peltier TE temperature controller, the sample was held at a constant 20 °C. The excitation wavelength was fixed at 295 nm, and emission spectra were recorded from 310 to 370 nm, with a bandwidth of 1 nm used for both excitation and emission beams. In the fluorometer stirred cuvette, DDM-solubilized LacY derivatives were suspended at 50 μM and equilibrated at 20 °C. Small volumes of detergent or lipid together with detergent were then added from various stock solutions of detergent micelles or mixed micelles. DDM micelles (50 mg/mL) containing either no lipid (DDM only), DOPC at 10 mg/mL or (9–10Br)-PC at 10 mg/mL or 20 mg/mL were incubated 2 h in the presence of 50 μM protein. Incorporation of phospholipid into the LacY-containing DDM micelles was measured by detecting the quenching of LacY intrinsic tryptophan fluorescence at 330 nm. The fluorescence extinction of Trp was evaluated using the ratio F/F_0_, where F and F_0_ are the fluorescence at 340 nm in the presence of mixed micelles containing or not containing brominated lipids, respectively. Results represent the average of five independent measurements obtained from emission spectra that were corrected for measurements with DDM only.

### Thermal denaturation followed by intrinsic tryptophan fluorescence spectroscopy

The fluorescence measurements were conducted using a QuantaMaster model QM3-SS (Photon Technology International) fluorescence spectrometer. Data were collected and analyzed using Felix 32 software. All measurements were conducted in degassed 50 mM Tris-HCl (pH 7.5)/100 mM NaCl/0.05% DDM. For thermal unfolding experiments, LacY derivatives containing all six native Trp residues were diluted to a final concentration of 10 μM. Using a Peltier TE temperature controller, the temperature gradient was set to an increase of 1 °C/min in a range from 20 °C to 90 °C. The excitation wavelength was fixed at 292 nm, and emission spectra were recorded from 310 to 370 nm, with a bandwidth of 1 nm used for both excitation and emission beams. Protein unfolding was measured by detecting the temperature-dependent changes in Trp fluorescence at emission wavelengths of 320 and 360 nm and temperature-dependent changes in the maximum emission wavelength (λ_max_). Data represents the average of three values obtained from emission spectra that were corrected for blank measurements.

### Circular dichroism spectropolarimetry

Circular dichroism experiments were conducted on a J-815 spectropolarimeter (Jasco, Tokyo, Japan) in a thermostated cell holder at 20 °C. Proteins were diluted in degassed 50 mM Tris-HCl (pH 7.5)/100 mM NaCl/0.05% DDM buffer 1 h before measurements. The scans were recorded using a bandwidth of 2 nm and an integration time of 1 s. For far-UV measurements, each spectrum was the average of 40 scans, with a scan rate of 100 nm/min, and with proteins at concentrations of 1 μM. Spectra in the presence of Sarge Unilamellar Vesicles (SUVs) were obtained using a lipid/protein molar ratio of 300. The spectra were corrected for the blank, were smoothed using the FFT filter (Jasco Software, Tokyo, Japan), and were treated as previously described^69^.

### Substrate binding assessment by fluorescence measurements

The fluorescence measurements were conducted using a QuantaMaster model QM3-SS (Photon Technology International) fluorescence spectrometer. Data were collected and analyzed using Felix 32 software. All measurements were conducted in degassed 50 mM Tris-HCl (pH 7.5)/100 mM NaCl/0.05% DDM. Steady-state measurements were carried out in 1 × 1 cm cuvettes (2 mL) with constant stirring. Emission spectra were recorded with a 1 nm bandwidth for both excitation and emission. All fluorescence changes were corrected for dilution resulting from addition of the ligand. Fluorescence changes were recorded using excitation and emission wavelengths of 295 and 330 nm for Trp → α-NPG FRET. Binding experiments were carried out by first equilibrating the protein sample with α-NPG. Displacement measurements were done by mixing protein pre-incubated with α-NPG with an excess of TDG (at 10 mM) containing the same concentration of α-NPG.

### Native agarose gel

Native-Agarose Gel Electrophoresis was carried out using a BioRad mini-sub cell DT unit. A 1% (w/v) horizontal SeaKem Gold agarose gel (10 cm × 6 cm × 5 mm) was prepared using native agarose gel buffer (25 mM Tris-HCl, 19.2 mM glycine, 0.05% DDM, pH 7.5). The horizontal gel was submerged in the apparatus containing native agarose gel buffer and electrophoresis was conducted at room temperature at 40 V for 4.5 h. Protein samples (10 μL at 1 mg/mL) were mixed with sample buffer (2.5 μL, native agarose gel buffer containing 30% (w/v) glycerol) and 5% (w/v) Coomassie blue G-250). Gels were stained in Imperial Protein Stain solution for 1 h and destained in water.

### Size-exclusion chromatography (SEC) experiments

Experiments were conducted at 4 °C using a Superdex 200 10/300 GL column (GE Healthcare) on an Äkta Purifier system (GE Healthcare). The column was equilibrated with 2 column volumes of 20 mM Tris (pH 7.4), 150 mM NaCl and 0.05% DDM. Before injection, purified protein samples (concentrations ranging from 0.25 to 0.4 mg/mL) were centrifuged at 100,000 g for 30 min at 4 °C. The sample was injected into the liquid chromatography system using a 250 μL loading loop. The flow rate was 0.5 mL/min during SEC and elution profiles were recorded at 280 nm.

### 96-well fluorescent based CPM thermostability assay

The thermostability assay was carried out as described by Stevens and co-workers^52^. The excitation wavelength was set at 387 nm, while the emission wavelength was 463 nm. One microliter of tested LacY derivative at 4 mg/mL was added to 150 μL of buffer containing 50 mM Tris-HCl (pH 7.5), 100 mM NaCl, detergent at three times the critical micellar concentration (CMC), and any other indicated additives in a 96-well black Nunc plate. The CPM dye at 4 mg/mL in DMSO was diluted 100-fold into buffer containing 50 mM Tris-HCl (pH 7.5), 100 mM NaCl, 0.05% DDM. After a 5 min incubation at room temperature to allow equilibration of the protein with the buffer components, 3 μL of CPM at 40 μg/mL was added, clear plate cover set in place, and within 5 min from protein addition fluorescence emission was measured at 463 nm (excitation 387 nm) on a BioTeK Synergy HT Microplate Reader instrument (BioTek Group). For temperature-dependent protein unfolding, assays were conducted with plates heated in a controlled way with a ramp rate of 1 °C/min in a range from 20 °C to 90 °C. Measurements were conducted every 30 s with 15 s shaking interval between each reading. For time-dependent protein unfolding, recordings were measured every 1 min for 3 h with 30 s shaking interval between each reading. The fraction of folded protein at each time point was calculated by the quotient of raw fluorescence measured at each time point divided by the maximal fluorescence measured for the detergent series.

### Two-dimensional crystallization and image processing

Purified LacY H205C/C154G in 0.05% DDM at a concentration ranging from 0.5 mg/mL to 2.5 mg/mL was mixed with various amounts of detergent-solubilized phospholipids. The lipid to protein ratios varied between 0.2 and 1 (w/w). The lipids used were 9:1 mixtures of DOPG:(18:1)CL, DOPG:DOPC, 1:1 or 2:1 mixtures of *E*. *coli* PG:*E*. *coli* CL and DOPG alone. Lipids were solubilized in 2% OG or a 1:1 (v/v) mixture of 2% OG and 0.2% DM. The mixtures were dialyzed against 10 mM Tris-HCl buffer (pH 5, 6.5 or 8) containing 0.02% (w/v) NaN_3_ in the presence or absence of monovalent salts (NaCl or KCl), divalent cations (MgCl_2_, CoCl_2_ or CaCl_2_), additives (glycerol, sucrose), substrate (10 mM TDG) at either 4 °C, room temperature or 37 °C, as indicated in Table 1 and Supplementary Table 1. Following dialysis, the crystallization buttons were opened. For negative staining, copper grids (G400, tedpella) were initially coated with colloidin film and a thin continuous layer of carbon was evaporated on the grids using carbon coater (BTT-IV evaporator, Denton). Samples were negatively stained on to the grids with 0.75% uranyl formate or 1% ammonium molybdate as described with modifications^70^. Samples were imaged using Joel 1400 transmission electron microscope operating at 120 kV (Morgagni M268, FEI, Hillsboro, OR) equipped with CCD camera (Need vendor info) or Polara 300-kV electron microscope (FEI Co.) equipped with a K2-summit direct electron detector (Gatan) at a magnification ranging from 10,000x to 32,000x.

### Data analysis and fitting

All data analysis and fitting were conducted using Prism 8 software (GraphPad Software Inc.). In all figures, solid lines indicate fits and error bars mark standard deviation (SD) for the measured parameters.

## Supplementary information

Supplementary information
