# Supplementary material for: The lipid-dependent structure and function of LacY can be recapitulated and analyzed in phospholipid-containing detergent micelles
Source: Sci Rep. 2019 Aug 5;9:11338. doi: 10.1038/s41598-019-47824-y (PMC6683142; doi:10.1038/s41598-019-47824-y)
Supplement: Supplementary file 1 — Supplementary information [file 41598_2019_47824_MOESM1_ESM.docx]

**The lipid-dependent structure and function of LacY can be recapitulated and analyzed in phospholipid-containing detergent micelles**

**Supplementary information**

Heidi Vitrac^1*^, Venkata Mallampalli^1^, Mikhail Bogdanov^1^ and William Dowhan^1^*

^1^Department of Biochemistry and Molecular Biology and the Center for Membrane Biology, University of Texas McGovern Medical School at Houston, Houston, TX, 77030

*Corresponding author: Heidi Vitrac, 6431 Fannin St., Suite 6.628, Department of Biochemistry and Molecular Biology, University of Texas McGovern Medical School at Houston, Houston, TX, 77030. Tel.: 713-500-6120; Fax: 713-500-0652; E-mail: Heidi.Vitrac@uth.tmc.edu

*Corresponding author: William Dowhan, 6431 Fannin St., Suite 6.200, Department of Biochemistry and Molecular Biology, University of Texas McGovern Medical School at Houston, Houston, TX, 77030. Tel.: 713-500-6051; Fax: 713-500-0652; E-mail: William.Dowhan@uth.tmc.edu

**Supplementary Table 1. Initial screen for two-dimensional crystallization of the inverted LacY involved determination of lipid to protein ratio (LPR), lipid species, and possible pH and additives that favor reconstitution and crystal formation.**

| **Lipids** | **[LacY]^a^** | **LPR** | **Divalent cations^b^** | **Monovalent ions** | **Other** | **Ligand^c^** | **T^d^** | **pH** | **Results** |
| --- | --- | --- | --- | --- | --- | --- | --- | --- | --- |
| DOPG | 1 | 0.75 | 200mM MgCl_2_ | 50mM NaCl | - | None | RT | 5 | Vesicles  Sheets  Stacks |
| DOPG | 1 | 0.75 | 200mM MgCl_2_ | 50mM NaCl | - | None | 4°C | 5 | Sheets  Stacks |
| DOPG | 1 | 0.75 | 200mM MgCl_2_ | 50mM NaCl | - | None | RT | 6.5 | Vesicles  Sheets  Stacks |
| DOPG | 1 | 0.75 | 200mM MgCl_2_ | 50mM NaCl | - | None | 4°C | 6.5 | Sheets  Stacks |
| DOPG | 1 | 0.75 | 200mM MgCl_2_ | 50mM NaCl | - | None | RT | 8 | Large Vesicles  Large sheets  Stacks |
| DOPG | 1 | 0.75 | 200mM MgCl_2_ | 50mM NaCl | - | None | 4°C | 8 | Large Vesicles  Large sheets  Stacks |
| DOPG | 1 | 0.75 | CoCl_2_ | 50mM NaCl | - | TDG | RT | 8 | Small Vesicles |
| DOPG | 1 | 0.75 | CoCl_2_ | 50mM NaCl | - | None | RT | 8 | Small Vesicles |
| DOPG | 1 | 0.75 | None | 50mM NaCl | - | TDG | RT | 8 | Reconstitution |
| DOPG | 1 | 0.75 | None | 50mM NaCl | - | None | RT | 8 | Reconstitution |
| DOPG | 1 | 1 | 10mM MgCl_2_  CoCl_2_ | 50mM NaCl | - | TDG | RT | 8 | Reconstitution |
| DOPG | 1 | 1 | CoCl_2_ | 50mM NaCl | - | TDG | RT | 8 | **Patchy crystal** |
| DOPG | 1 | 1 | CoCl_2_ | 50mM NaCl | - | None | RT | 8 | *Aggregation* |
| DOPG | 2.21 | 1 | 50mM MgCl_2_ | 50mM NaCl | - | None | RT | 8 | *Aggregation* |
| DOPG | 2.21 | 1 | 50mM MgCl_2_ | 50mM NaCl | 2% sucrose | None | RT | 8 | *Aggregation* |
| DOPG | 2.21 | 1 | 50mM MgCl_2_ | 50mM NaCl | 2% glycerol | None | RT | 8 | *Aggregation* |
| DOPG | 2.21 | 1 | 50mM MgCl_2_ | 50mM NaCl | - | None | RT | 6 | *Aggregation* |
| DOPG | 2.21 | 1 | 50mM MgCl_2_ | 50mM NaCl | 2% sucrose | None | RT | 6 | Sheets |
| DOPG | 2.21 | 1 | 50mM MgCl_2_ | 50mM NaCl | 2% glycerol | None | RT | 6 | Sheets |
| DOPG | 2.21 | 1 | 50mM MgCl_2_ | 50mM NaCl | - | None | RT | 8 | *Aggregation*  Vesicles |
| DOPG | 2.21 | 1 | 50mM MgCl_2_ | 50mM NaCl | - | None | RT | 6 | *Aggregation*  Large Vesicles |
| DOPG | 2.5 | 0.75 | CoCl_2_ | 50mM NaCl | - | TDG | RT | 8 | Small Vesicles |
| DOPG | 2.5 | 0.75 | CoCl_2_ | 50mM NaCl | - | None | RT | 8 | Sheets |
| DOPG | 2.5 | 0.75 | None | 50mM NaCl | - | TDG | RT | 8 | *Aggregation*  Very small vesicles |
| DOPG | 2.5 | 0.75 | None | 50mM NaCl | - | None | RT | 8 | Very small vesicles  Sheets |
| DOPG | 2.5 | 0.75 | None | 50mM NaCl | - | TDG | RT | 8 | *Aggregation* |
| DOPG | 2.5 | 0.75 | None | 50mM NaCl | - | None | RT | 8 | *Aggregation*  Large Vesicles |
| DOPG | 2.5 | 0.75 | None | 50mM NaCl | - | TDG | RT | 8 | Sheets |
| DOPG | 2.5 | 0.75 | None | 50mM NaCl | - | None | RT | 8 | *Aggregation*  Reconstitution |
| DOPG | 2.5 | 1 | CoCl_2_ | 50mM NaCl | - | None | RT | 8 | *Aggregation*  Small Vesicles |
| DOPG | 2.5 | 1 | None | 50mM NaCl | - | TDG | RT | 8 | *Aggregation* |
| DOPG | 2.5 | 1 | None | 50mM NaCl | - | None | RT | 8 | **Patchy Crystals**  Large vesicles  Sheets |
| DOPG | 2.5 | 1 | CoCl_2_ | 50mM NaCl | - | TDG | RT | 8 | **Patchy crystals**  Vesicles  Sheets |
| DOPG/CL(18:1) 9:1 | 1 | 0.75 | 200mM MgCl_2_  CaCl_2_ | 50mM NaCl | - | None | RT | 8 | Sheets |
| DOPG/CL(18:1) 9:1 | 1 | 0.75 | 200mM MgCl_2_  CaCl_2_ | 500mM LiCl | - | None | RT | 8 | Sheets  Stacks |
| DOPG/CL(18:1) 9:1 | 1 | 0.75 | 200mM MgCl_2_  CaCl_2_ | 500mM KCl | - | None | RT | 8 | Large Sheets  Stacks |
| DOPG/CL(18:1) 9:1 | 1 | 0.75 | 200mM MgCl_2_  CaCl_2_ | 500mM NaCl | - | None | RT | 8 | Large Sheets  Stacks |
| DOPG/CL(18:1) 9:1 | 1 | 0.75 | 200mM MgCl_2_  CaCl_2_ | 50mM LiCl | - | None | 37°C | 8 | Sheets  Stacks |
| DOPG/CL(18:1) 9:1 | 1 | 0.75 | 200mM MgCl_2_  CaCl_2_ | 50mM NaCl | - | None | 37°C | 8 | Large Sheets  Stacks |
| DOPG/CL(18:1) 9:1 | 1 | 0.75 | 200mM MgCl_2_  CaCl_2_ | 500mM LiCl | - | None | 37°C | 8 | Large Sheets  Stacks |
| DOPG/CL(18:1) 9:1 | 1 | 0.75 | 200mM MgCl_2_  CaCl_2_ | 500mM KCl | - | None | 37°C | 8 | Large Sheets  Stacks |
| DOPG/CL(18:1) 9:1 | 1 | 0.75 | 200mM MgCl_2_  CaCl_2_ | 500mM NaCl | - | None | 37°C | 8 | - |
| DOPG/CL(18:1) 9:1 | 1 | 0.75 | 200mM MgCl_2_  CaCl_2_ | - | - | None | RT | 8 | **Small Patchy Crystals**  Sheets  Stacks |
| DOPG/CL(18:1) 9:1 | 1 | 0.75 | 200mM MgCl_2_  CaCl_2_ | 50mM LiCl | - | None | RT | 8 | **Small Patchy Crystals**  Large Sheets  Stacks |
| DOPG/CL(18:1) 9:1 | 1 | 0.75 | 200mM MgCl_2_  CaCl_2_ | 50mM KCl | - | None | RT | 8 | Sheets  Stacks |
| DOPG/CL(18:1) 9:1 | 1 | 0.75 | 200mM MgCl_2_  CaCl_2_ | - | - | None | 37°C | 8 | **Patchy Crystals**  Large Sheets  Stacks |
| DOPG/CL(18:1) 9:1 | 1 | 0.75 | 200mM MgCl_2_  CaCl_2_ | 50mM KCl | - | None | 37°C | 8 | **Patchy Crystals**  Large Sheets  Stacks |
| DOPG/DOPC 9:1 | 1 | 0.75 | 200mM MgCl_2_ | - | - | None | RT | 5 | Vesicles |
| DOPG/DOPC 9:1 | 1 | 0.75 | 200mM MgCl_2_ | - | - | None | RT | 7 | Vesicles  Sheets  Stacks |
| DOPG/DOPC 9:1 | 1 | 0.75 | 200mM MgCl_2_ | - | - | None | 4°C | 7 | Sheets  Stacks |
| DOPG/DOPC 9:1 | 1 | 0.75 | 200mM MgCl_2_ | - | - | None | RT | 8 | Large Vesicles |
| DOPG/DOPC 9:1 | 1 | 0.75 | None | 50mM NaCl | - | TDG | RT | 8 | Very small vesicles |
| DOPG/DOPC 9:1 | 1 | 0.75 | None | 50mM NaCl | - | None | RT | 8 | Reconstitution |
| DOPG/DOPC 9:1 | 1 | 0.75 | 200mM MgCl_2_ | - | - | None | 4°C | 5 | **Patchy Crystals**  Large vesicles  Sheets |
| DOPG/DOPC 9:1 | 1 | 0.75 | 200mM MgCl_2_ | - | - | None | 4°C | 8 | **Patchy Crystals**  Large vesicles  Sheets |
| DOPG/DOPC 9:1 | 1 | 1 | 10mM MgCl_2_, CoCl_2_ | 50mM NaCl | - | TDG | RT | 8 | *Aggregation* |
| DOPG/DOPC 9:1 | 1 | 1 | CoCl_2_ | 50mM NaCl | - | TDG | RT | 8 | Vesicles |
| DOPG/DOPC 9:1 | 1 | 1 | CoCl_2_ | 50mM NaCl | - | None | RT | 8 | *Aggregation* |
| DOPG/DOPC 9:1 | 2.5 | 0.75 | None | 50mM NaCl | - | TDG | RT | 8 | Vesicles |
| DOPG/DOPC 9:1 | 2.5 | 1 | 10mM MgCl_2_  CoCl_2_ | 50mM NaCl | - | None | RT | 8 | *Aggregation*  Small Vesicles |
| DOPG/DOPC 9:1 | 2.5 | 1 | CoCl_2_ | 50mM NaCl | - | TDG | RT | 8 | Very small vesicles |
| DOPG/DOPC 9:1 | 2.5 | 1 | CoCl_2_ | 50mM NaCl | - | None | RT | 8 | *Aggregation*  Sheets |
| PG/CL 1:1 | 0.5 | 0.2 | 200mM MgCl_2_ | - | - | None | RT | 8 | Sheets  Stacks |
| PG/CL 1:1 | 0.5 | 0.5 | 200mM MgCl_2_ | - | - | None | RT | 8 | Sheets  Stacks |
| PG/CL 1:1 | 0.5 | 0.75 | 200mM MgCl_2_ | - | - | None | RT | 8 | **Patchy Crystals**  *Aggregation*  Vesicles  Stacks |
| PG/CL 1:1 | 1 | 0.2 | 200mM MgCl_2_ | - | - | None | RT | 8 | Sheets |
| PG/CL 1:1 | 1 | 0.2 | 50mM MgCl_2_ | - | - | None | RT | 8 | *Aggregation*  Large Vesicles  Sheets |
| PG/CL 1:1 | 1 | 0.2 | 50mM MgCl_2_ | - | 20% glycerol | None | RT | 8 | Sheets  Stacks |
| PG/CL 1:1 | 1 | 0.2 | 50mM MgCl_2_ | - | 20% glycerol | None | RT | 8 | **Patchy Crystals**  *Aggregation*  Vesicles |
| PG/CL 1:1 | 1 | 0.5 | 200mM MgCl_2_ | - | - | None | RT | 8 | Sheets  Stacks |
| PG/CL 1:1 | 1 | 0.5 | 50mM MgCl_2_ | - | - | None | RT | 8 | Sheets  Stacks |
| PG/CL 1:1 | 1 | 0.5 | 50mM MgCl_2_ | - | 20% glycerol | None | RT | 8 | Sheets  Stacks |
| PG/CL 1:1 | 1 | 0.5 | 50mM MgCl_2_ | - | 20% glycerol | None | RT | 8 | **Patchy Crystals**  *Aggregation*  Vesicles |
| PG/CL 1:1 | 1 | 0.75 | 200mM MgCl_2_ | - | - | None | RT | 8 | Vesicles |
| PG/CL 1:1 | 1 | 0.75 | 50mM MgCl_2_ | - | - | None | RT | 8 | Sheets |
| PG/CL 1:1 | 1 | 0.75 | 50mM MgCl_2_ | - | 20% glycerol | None | RT | 8 | *Aggregation*  Large Vesicles  Sheets |
| PG/CL 1:1 | 1 | 0.75 | 50mM MgCl_2_ | - | 20% glycerol | None | RT | 8 | Sheets  Stacks |
| PG/CL 1:1 | 1 | 1 | 200mM MgCl_2_ | - | - | None | RT | 8 | - |
| PG/CL 1:1 | 1 | 1 | 50mM MgCl_2_ | - | - | None | RT | 8 | Sheets  Stacks |
| PG/CL 1:1 | 1 | 1 | 50mM MgCl_2_ | - | 20% glycerol | None | RT | 8 | Vesicles  Sheets  Stacks |
| PG/CL 1:1 | 1 | 1 | 50mM MgCl_2_ | - | 20% glycerol | None | RT | 8 | - |
| PG/CL 2:1 | 1 | 0.2 | 200mM MgCl_2_ | - | - | None | RT | 8 | Sheets  Stacks |
| PG/CL 2:1 | 1 | 0.2 | 50mM MgCl_2_ | - | - | None | RT | 8 | - |
| PG/CL 2:1 | 1 | 0.2 | 50mM MgCl_2_ | - | 20% glycerol | None | RT | 8 | Vesicles  Sheets |
| PG/CL 2:1 | 1 | 0.2 | 50mM MgCl_2_ | - | 20% glycerol | None | RT | 8 | **Patchy Crystals**  *Aggregation*  Vesicles  Stacks |
| PG/CL 2:1 | 1 | 0.5 | 200mM MgCl_2_ | - | - | None | RT | 8 | Sheets  Stacks |
| PG/CL 2:1 | 1 | 0.5 | 50mM MgCl_2_ | - | - | None | RT | 8 | - |
| PG/CL 2:1 | 1 | 0.5 | 50mM MgCl_2_ | - | 20% glycerol | None | RT | 8 | Sheets  Stacks |
| PG/CL 2:1 | 1 | 0.5 | 50mM MgCl_2_ | - | 20% glycerol | None | RT | 8 | **Patchy Crystals**  *Aggregation*  Vesicles  Stacks |
| PG/CL 2:1 | 1 | 0.75 | 200mM MgCl_2_ | - | - | None | RT | 8 | Sheets |
| PG/CL 2:1 | 1 | 0.75 | 50mM MgCl_2_ | - | - | None | RT | 8 | - |
| PG/CL 2:1 | 1 | 0.75 | 50mM MgCl_2_ | - | 20% glycerol | None | RT | 8 | Sheets  Stacks |
| PG/CL 2:1 | 1 | 0.75 | 50mM MgCl_2_ | - | 20% glycerol | None | RT | 8 | *Aggregation* |
| PG/CL 2:1 | 1 | 1 | 50mM MgCl_2_ | - | - | None | RT | 8 | Sheets  Stacks |
| PG/CL 2:1 | 1 | 1 | 50mM MgCl_2_ | - | 20% glycerol | None | RT | 8 | *Aggregation*  Sheets |
| PG/CL 2:1 | 1 | 1 | 50mM MgCl_2_ | - | 20% glycerol | None | RT | 8 | *Aggregation*  Sheets  Stacks |
| PG/CL 2:1 | 1 | 1 | 200mM MgCl_2_ | - | - | None | RT | 8 | **Patchy Crystals**  *Aggregation*  Vesicles  Stacks |

^a^ LacY concentration in mg/mL

^b^ CoCl_2_ and CaCl_2_ at 10 mM

^c^ TDG at 10 mM

^d^ Temperature at which the dialysis is conducted


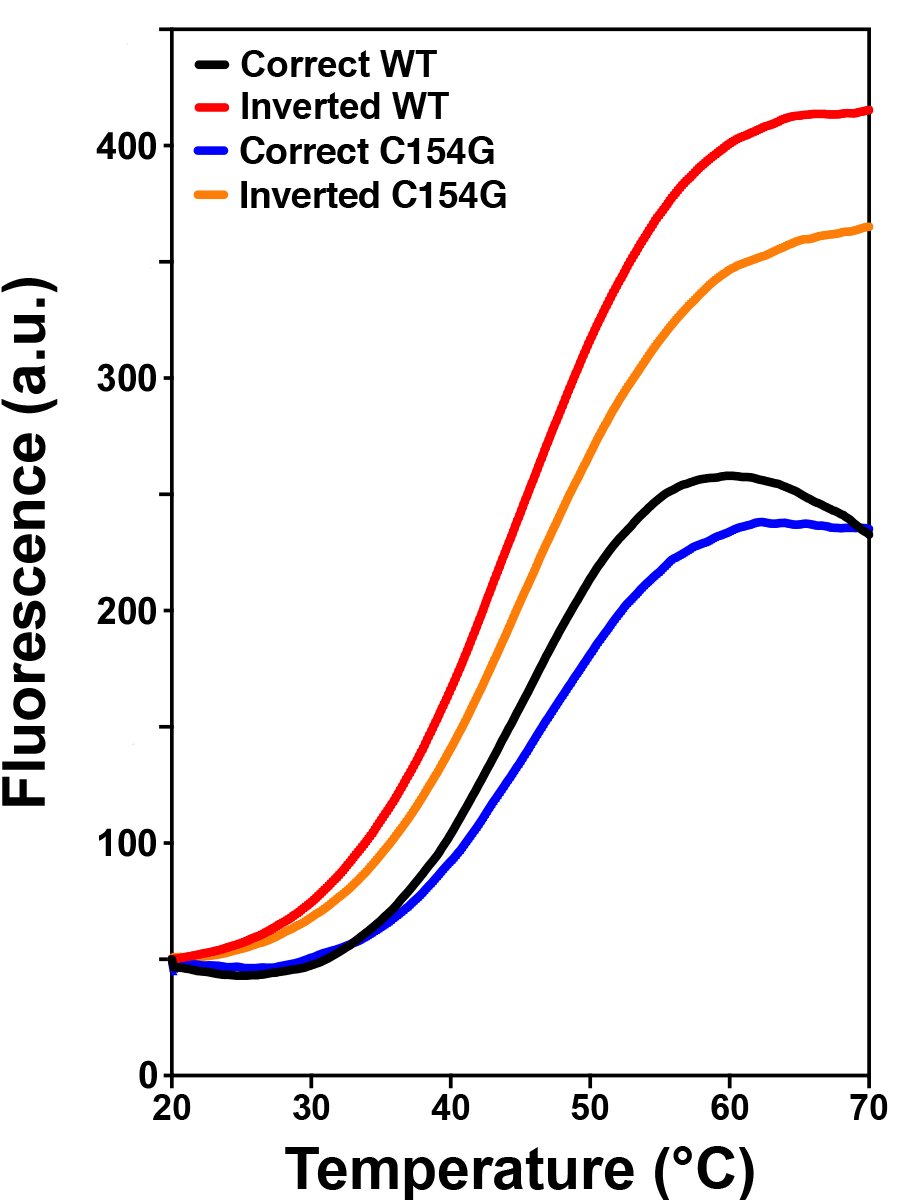


**Supplemental Figure 1.** **The C154G mutation improves the stability of LacY in DDM micelles in both the correct and the inverted topology.** Representative melting curves using the CPM dye binding assay for WT and C154G LacY isolated from PE-containing (Correct) and PE-lacking (inverted) cells. LacY was diluted to 26 μg/mL in 50 mM Tris-HCl (pH 7.5), 100 mM NaCl containing 0.05% DDM.

**
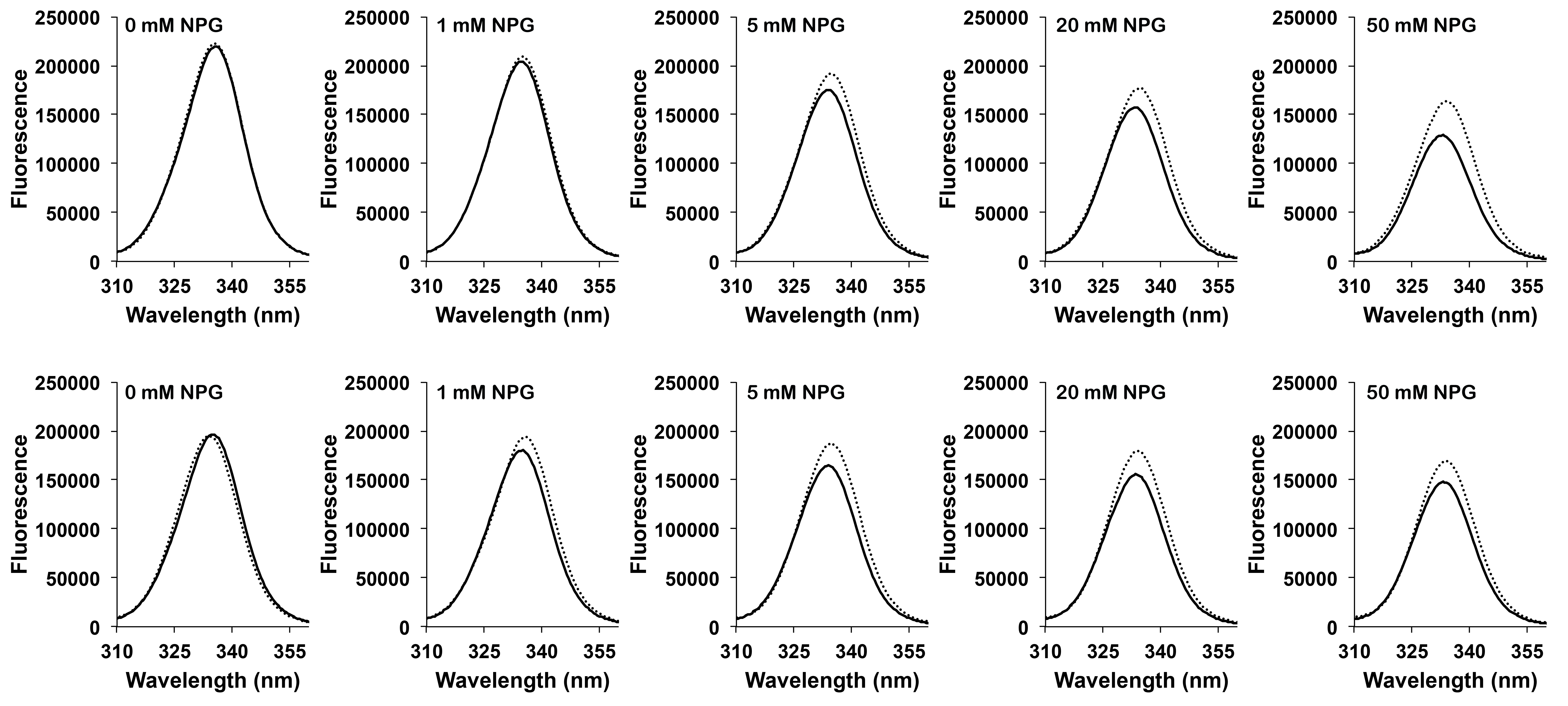
**

**Supplemental Figure 2. Binding of α-NPG to C154G LacY as detected by Trp→α-NPG FRET.** Measurements were carried out in 50 mM Tris-HCl (pH 7.5), 100 mM NaCl containing 0.05% DDM at a protein concentration of 5 μM; excitation was at 295 nm. Trp emission spectra at different concentrations of **α**-NPG are depicted. Solid lines represent Trp fluorescence at increasing **α**-NPG concentrations; broken lines indicate spectra after addition of 10 mM TDG. Results of experiments conducted on LacY purified from PE-containing or PE-lacking cells are depicted in the top or bottom row, respectively.


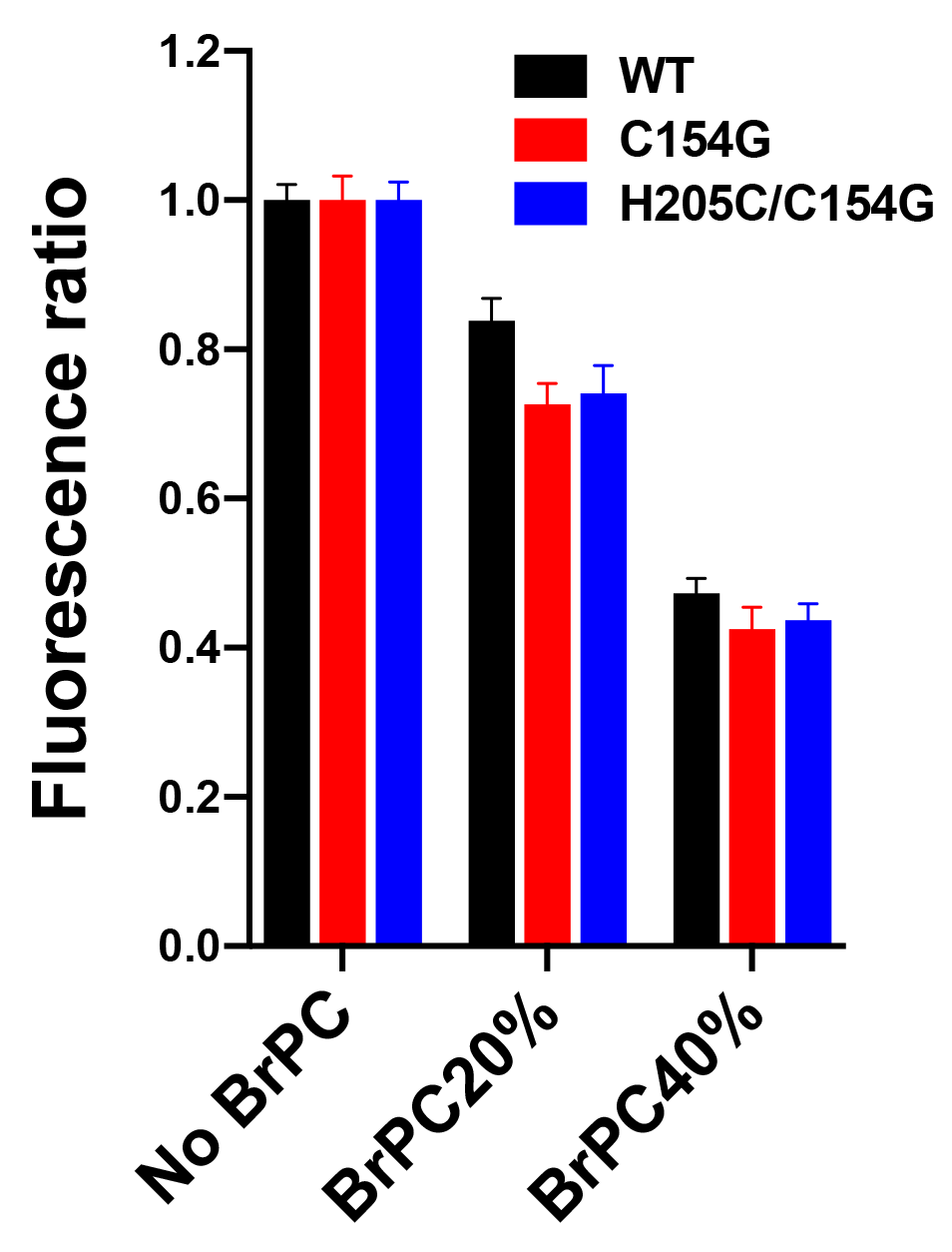


**Supplemental Figure 3. Phospholipids can be incorporated in LacY-containing DDM micelles after LacY purification**. Intrinsic fluorescence extinction of Trp of three LacY templates isolated from PE-lacking cells and solubilized in DDM induced by the resupply of increasing amounts of brominated lipids.


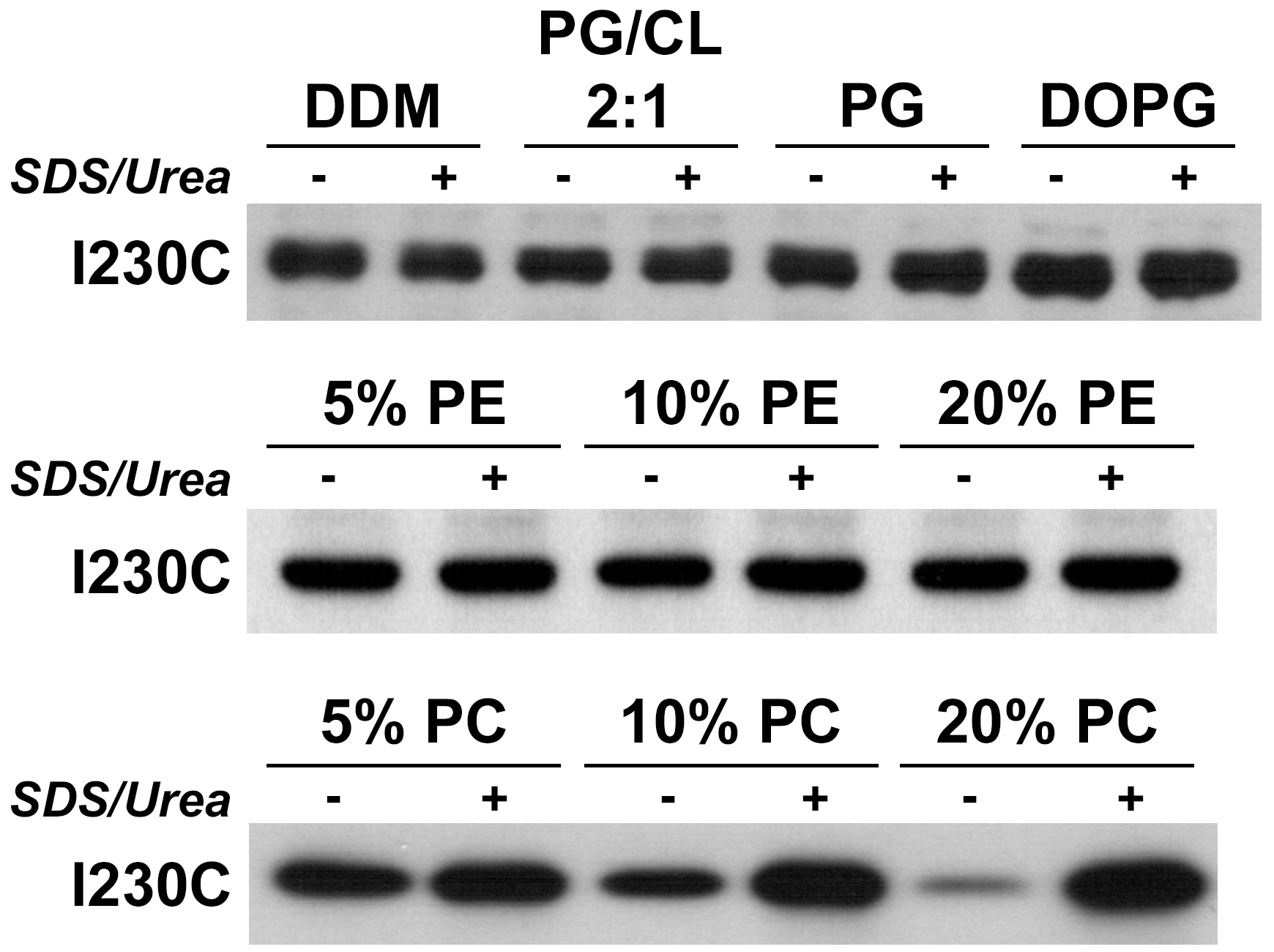


**Supplemental Figure 4. Determination of TMD VII exposure to the aqueous environment using the SCAM^TMD^ assay after resupply of phospholipids to the LacY-containing DDM micelles.** Cysteine-less LacY with a single-cysteine replacement in TMD VII (I230C) was purified from PE-lacking cells and solubilized in DDM. Resupply of phospholipids was conducted prior to MPB labeling by incubation with DDM-solubilized phospholipids. After treatment with MPB, samples were subjected to SCAM^TMD^ as described in Methods. Representative results (cropped western blots) are shown for the TMD VII domain in LacY subjected incubation with DDM micelles containing either no lipids (DDM) or a 2:1 mixture of *E. coli* PG and CL, *E. coli* PG, DOPG, 5, 10 or 20% DOPE and 5, 10 or 20% of DOPC.


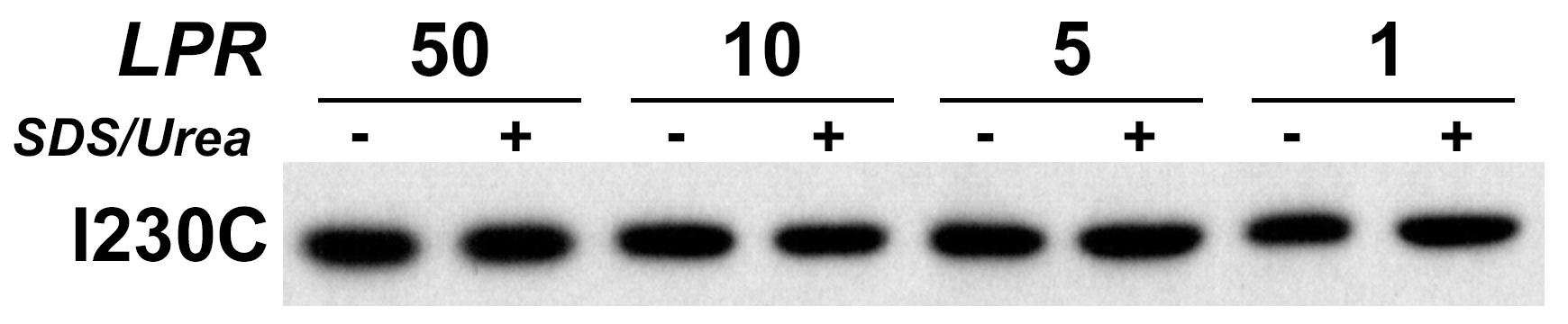


**Supplemental Figure 5. Determination of TMD VII exposure to the aqueous environment using the SCAM^TMD^ assay after proteoliposomes reconstitution at various Lipid-to-Protein ratios using DOPG.** LacY with a single-cysteine replacement in TMD VII (I230C) was purified from PE-lacking cells and solubilized in DDM. Reconstitution in proteoliposomes was conducted as described under 2D crystallization screening. After recovery of proteoliposomes by centrifugation and treatment with MPB, samples were subjected to SCAM^TMD^ as described in Methods. Representative results (cropped western blots) are shown for LPR = 1, 5, 10 and 50.
